# ROR1-STAT3 signaling contributes to ovarian cancer intra-tumor heterogeneity

**DOI:** 10.1038/s41420-023-01527-6

**Published:** 2023-07-03

**Authors:** Emilia Piki, Alice Dini, Juuli Raivola, Kari Salokas, Kaiyang Zhang, Markku Varjosalo, Teijo Pellinen, Katja Välimäki, Kristina Tabor Veskimäe, Synnöve Staff, Sampsa Hautaniemi, Astrid Murumägi, Daniela Ungureanu

**Affiliations:** 1 Disease Networks Unit, Faculty of Biochemistry and Molecular Medicine, University of Oulu, 90014 Oulu, Finland; 2 Applied Tumor Genomics, Research Program Unit, Faculty of Medicine, University of Helsinki, 00014 Helsinki, Finland; 3 Institute of Biotechnology, University of Helsinki, 00014 Helsinki, Finland; 4 Research Program in Systems Oncology, Research Program Unit, Faculty of Medicine, University of Helsinki, 00014 Helsinki, Finland; 5 Institute for Molecular Medicine Finland, FIMM, Helsinki Institute of Life Science (HiLIFE) University of Helsinki, 00014 Helsinki, Finland; 6 Department of Obstetrics and Gynecology, Tampere University Hospital and Faculty of Medicine and Health Technology, Tampere University, 33014 Tampere, Finland

**Keywords:** Ovarian cancer, Tumour heterogeneity

## Abstract

Wnt pathway dysregulation through genetic and non-genetic alterations occurs in multiple cancers, including ovarian cancer (OC). The aberrant expression of the non-canonical Wnt signaling receptor ROR1 is thought to contribute to OC progression and drug resistance. However, the key molecular events mediated by ROR1 that are involved in OC tumorigenesis are not fully understood. Here, we show that ROR1 expression is enhanced by neoadjuvant chemotherapy, and Wnt5a binding to ROR1 can induce oncogenic signaling via AKT/ERK/STAT3 activation in OC cells. Proteomics analysis of isogenic ROR1-knockdown OC cells identified STAT3 as a downstream effector of ROR1 signaling. Transcriptomics analysis of clinical samples (*n* = 125) revealed that ROR1 and STAT3 are expressed at higher levels in stromal cells than in epithelial cancer cells of OC tumors, and these findings were corroborated by multiplex immunohistochemistry (mIHC) analysis of an independent OC cohort (*n* = 11). Our results show that ROR1 and its downstream STAT3 are co-expressed in epithelial as well as stromal cells of OC tumors, including cancer-associated fibroblasts or CAFs. Our data provides the framework to expand the clinical utility of ROR1 as a therapeutic target to overcome OC progression.

## Introduction

OC is ranked second in the list of gynecological cancer incidence [1]. Often due to advanced-stage diagnosis, patients with OC have a very poor 5-year survival rate [2, 3]. The most prevalent subtype is high-grade serous ovarian cancer (HGSOC, 75–80% of cases), which is defined by a nearly 100% p53 mutation rate, defects in homologous recombination (HR) repair, mutations in BRCA1/2 and extensive copy number aberrations [4]. HGSOC is also highly heterogeneous due to the polyclonal composition of single tumors arising from different subclones [5, 6]. Single-cell transcriptomic analyses have shown that HGSOC tumors are composed of various cell populations, with epithelial cancer cells and cancer-associated stroma as the main cellular subtypes [6]. Our recent study also identified that tumor heterogeneity is tissue and patient-specific, and tumor diversity is acquired early during HGSOC evolution [6]. Although HGSOC patients are initially responsive to platinum-based chemotherapy, chemoresistance occurs in almost 80% of cases [3]. This highlights an unmet need for novel clinical treatments, as there are very few approved targeted therapies for this cancer.

Wnt-pathway was identified as one of the six significantly enriched cancer pathways in the transcriptomic analysis of DECIDER clinical samples, along with NOTCH, PI3K/AKT, MAPK, and ERBB pathways [6]. In healthy cells, the Wnt pathway helps to control the balance between self-renewal and differentiation, and the disruption of these processes can lead to cancer [7]. Genetic alterations are commonly identified in genes encoding for proteins participating in the canonical Wnt signaling, whereas cancer-associated hotspot mutations in the non-canonical Wnt pathway are less common, affecting the pharmacological approaches to target its potential effectors. The best strategies thus far have been to target the non-canonical Wnt signaling in distinct tumor subclasses that show reliance on this pathway for survival and progression [8–10]. For instance, the therapeutic relevance of targeting the non-canonical Wnt receptors ROR1 and ROR2 (receptor tyrosine kinase-like orphan receptor) has been investigated in numerous cancers of both hematological and epithelial origin [11, 12]. Both ROR1 and ROR2 belong to the same ROR family of receptors that are capable of binding Wnt5a/b and transducing signaling within the non-canonical Wnt pathway [11]. In OC, the activation of ROR1/ROR2 signaling has been linked to processes driving tumorigenesis such as cell proliferation, invasion, and drug resistance [13–15]. Moreover, previous data have indicated that high ROR1 expression is associated with poor overall survival (OS) and disease-free survival (DFS) of OC patients, whereas the expression of ROR2 in OC has been linked to the development of chemoresistance [16, 17]. In particular, we demonstrated that glucocorticoid-mediated ROR1 upregulation in OC could promote cancer stem-cell phenotype and drug resistance [18].

The selective expression of ROR1, mainly attributed to malignant cells, has made this receptor an attractive target for cancer therapy to avoid off-target cytotoxic effects [15]. However, the development of more specific anti-ROR1 antibodies has shown that this receptor is also expressed in normal human tissues such as parathyroid, pancreatic islets, and differentiated adipocytes, as well as in tumor-associated stroma cells [13, 19]. In cancer cells, ROR1 cooperates in pro-survival pathways such as PI3K/AKT, EGFR, and MET signaling to enhance tumor cell growth and survival, EMT (epithelial–mesenchymal transition), and metastasis [18, 20–22]. However, the molecular mechanisms by which ROR1 expression contributes to OC progression are yet to be fully understood.

Here, we investigated the molecular mechanisms associated with ROR1 expression in HGSOC. Through computational integration of clinical data (DECIDER cohort, *n* = 125 [6]), we observed that ROR1 and Wnt5b (but not ROR2, nor Wnt5a) expression is upregulated in post-NACT (post-neoadjuvant chemotherapy) clinical samples. Proteomics analysis of isogenic OC cells following ROR1 knockdown identified STAT3 as a downstream effector of ROR1 signaling. We also show that binding of Wnt5a to ROR1 activates the pro-survival AKT/ERK and STAT3 pathways in OC cells, corroborating previous findings reporting that ROR1 could induce oncogenic signaling via the PI3K/AKT/ERK axis [20]. Interestingly, we observed high Wnt5a/ROR1/STAT3 transcriptomic levels in OC stromal cells, and these findings were corroborated by multiplex immunohistochemistry (mIHC) analysis of an independent OC patient cohort (*n* = 11). Importantly, ROR1/STAT3 expression was also detected in cancer-associated fibroblasts or CAFs, which are often associated with worse disease prognosis in many cancers [23]. Finally, we uncovered a common gene-expression signature in ROR1^high^/STAT3^high^ clinical samples, highlighting the inflammatory response pathways, transcriptional regulation of adipocyte differentiation, and adipogenesis as the main biological processes mediated by these genes. Together, our work reveals a new role for ROR1/STAT3 signaling in tumor heterogeneity and inflammation in OC.

## Results

### ROR1 expression is enhanced in post-NACT OC clinical samples

**Fig. 1:**
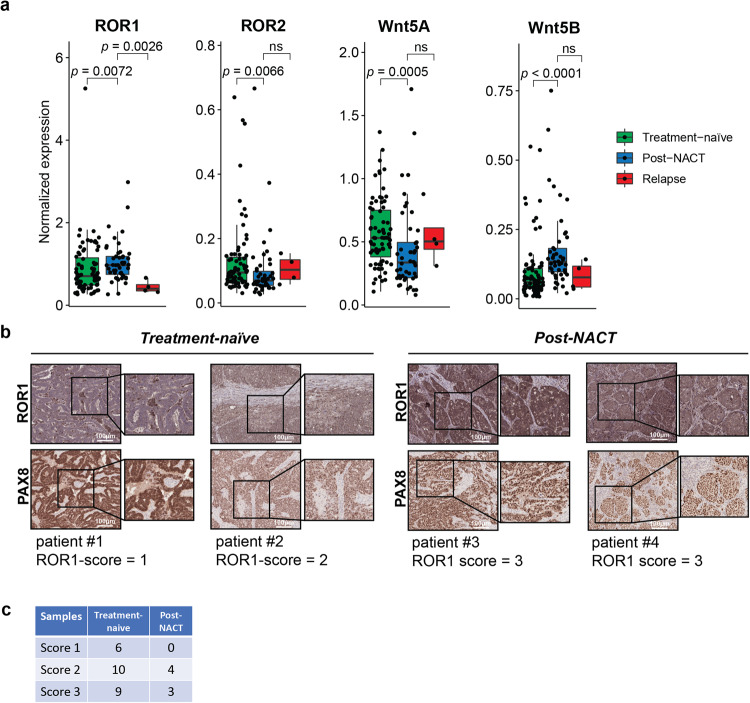
ROR1 expression is enhanced by NACT in HGSOC samples. **a** Boxplots representing the expression levels of ROR1, ROR2, Wnt5a, and Wnt5b in treatment-naïve (*n* = 75), post-NACT (*n* = 46), and relapsed (*n* = 4) HGSOC patient samples in bulk RNA-seq data of the epithelial ovarian cancer cells from the DECIDER cohort. The significance was calculated with the Wilcoxon rank-sum test. ns, not significant (*p* > 0.05). **b** Representative IHC images (*n* = 32) of treatment-naïve (patients #1 and #2) and post-NACT samples (patients #3 and #4) showing ROR1 and PAX8 levels. The clinicopathological classification of the samples based on their ROR1 IHC staining intensity (score 0–3) is reported under the sample names. ROR1 intensity was evaluated on PAX8 positive areas. Boxes show the area magnified on the right. Scale bar = 100 µm. **c** Table summary showing the treatment status and ROR1 scores evaluated by IHC (*n* = 32).

First, we investigated how NACT modulates the expression of Wnt5-RORs signaling in OC by transcriptomics and immunohistochemistry (IHC) analysis. We assessed the expression of non-canonical Wnt receptors ROR1/ROR2 and their ligands Wnt5a/Wnt5b in the epithelial ovarian cancer cell-specific transcriptomic dataset of the DECIDER cohort comprising 125 clinically defined HGSOC patient samples (Suppl. Fig. 1a). Our analysis identified higher ROR1 (*p* = 0.0072) and Wnt5b (*p* < 0.0001) but lower ROR2 (*p* = 0.0066) and Wnt5a (*p* = 0.0005) levels in post-NACT (*n* = 46) samples compared to treatment-naïve (*n* = 75) or relapsed (*n* = 4) samples (Fig. 1a). These findings are in line with previous studies showing that glucocorticoids such as dexamethasone, which is often used during NACT, enhances ROR1 expression in breast cancer and OC [18, 24]. We also performed IHC analysis of ROR1 protein expression in a retrospective OC cohort comprising patient samples collected before (treatment-naïve, *n* = 25) and after NACT (post-NACT, *n* = 7, Suppl. Table 1, Suppl. Fig. 1b). All tumors tested positive for paired box gene 8 (PAX8) staining, an established marker for epithelial OC cells [25]. The clinicopathological analysis of ROR1 IHC staining was evaluated from low (score 1, ROR1^low^) to high (score 3, ROR1^high^) based on the overall staining intensity and distribution. Accordingly, we identified strong ROR1 levels in 37.5% of samples (*n* = 12, ROR1^high^) and low ROR1 levels in 18.7% samples (*n* = 6, ROR1^low^, Fig. 1b), whereas 43% of samples (*n* = 14) showed a staining distribution in between these levels (score 2). Of the seven post-NACT samples, three were score 3 (ROR1^high^), and four corresponded to score 2, whereas ROR1^low^ samples were identified only in the treatment-naïve cohort, corroborating our transcriptomics data that ROR1 expression is enhanced in post-NACT OC samples (Fig. 1c).

### STAT3 is a downstream target of ROR1 in OC cells

**Fig. 2:**
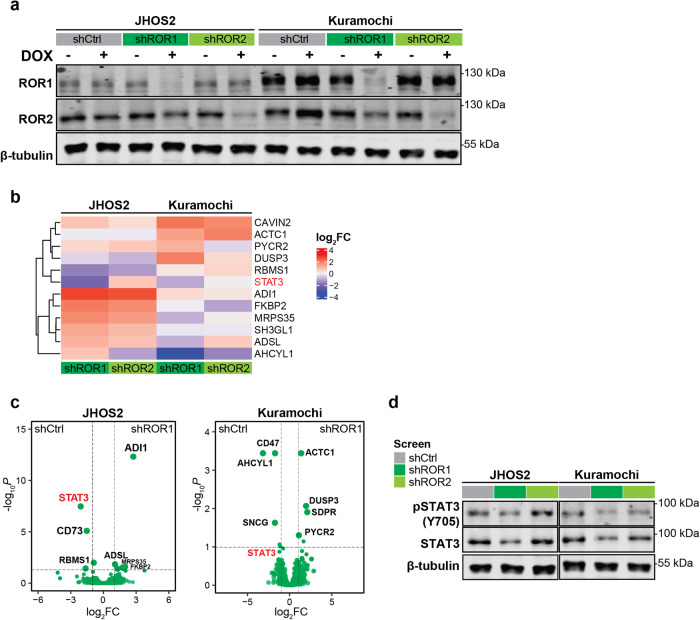
STAT3 is a downstream transcription factor for ROR1 in OC cells. **a** Immunoblotting analysis of untreated or DOX-treated (4 days) JHOS2 and Kuramochi shRNA cell lysates showing the efficient downregulation of ROR1 and ROR2. **b** Heatmap of selected differentially expressed proteins of shCtrl vs. shROR1 or shROR2 samples in JHOS2 or Kuramochi cell lines. **c** Volcano plots showing the distribution of fold changes and (adjusted) *p*-values for the comparisons of differentially expressed proteins in shROR1 vs. shCtrl samples in JHOS2 and Kuramochi cell lines (*p* < 0.05). **d** Immunoblot analysis of DOX-treated (four days) JHOS2 and Kuramochi shRNA cell lysates showing pSTAT3 and STAT3 expression. For **a** and **d**, β-tubulin was used as a loading control, and a representative of three independent experiments is shown.

To identify downstream ROR1/ROR2 signaling effectors in OC cells, we used JHOS2 and Kuramochi cell lines that express endogenous levels of ROR1 and ROR2 to perform doxycycline (DOX)-inducible stable shRNA knockdown for each receptor. Two different shRNAs per target were tested along with control (scrambled, shCtrl) to identify the most efficient knockdown for further analysis (Suppl. Fig. 2). Immunoblotting analysis of cell lysates showed efficient knockdown of ROR1 or ROR2 only in DOX-treated isogenic cell lines (Fig. 2a, Suppl. Fig. 2). Next, we performed liquid chromatography-tandem mass spectrometry (LC–MS/MS) of JHOS2 and Kuramochi shRNA isogenic cell lines. Comparison of differentially expressed proteins (log_2_FC, *p* < 0.05; Fig. 2b) of each shROR1 and shROR2 sample with the shCtrl sample of the same cell line identified 12 proteins representing diverse molecular effectors such as CAVIN2 (caveolae associated protein-2) from the caveolae-family, DUSP3 (dual specificity phosphatase 3) from MAPK-signaling, ATCT1 (actin alpha cardiac muscle 1) involved in cell motility, and several other proteins involved in various biosynthesis and RNA-processing pathways (Suppl. Table 2). Interestingly, we observed STAT3 downregulation in both JHOS2 and Kuramochi shROR1 isogenic cells (but not shROR2 cells, Fig. 2c), and this finding was confirmed by immunoblotting analysis of both shROR1 samples (Fig. 2d). Our findings are in line with previous data showing that STAT3 is modulated by ROR1 expression. However, this mechanism was previously identified in hematological [26, 27] and gastric cancers [28].

### Wnt5a-binding to ROR1 activates pERK/pAKT/pSTAT3 signaling in OC cells

Aberrant Wnt5a signaling emerged as an important event in tumorigenesis, where Wnt5a expression was identified in metastatic OC samples and in ascites isolated from OC patients [29, 30]. Previously, we have shown that Wnt5a binding to ROR1 activates pERK/pAKT intracellular signaling in ROR1-positive murine interleukin-3 (IL-3)-dependent pro-B (BaF3) isogenic clones [20], therefore, we asked whether a similar effect is mediated by Wnt5a in OC.

**Fig. 3:**
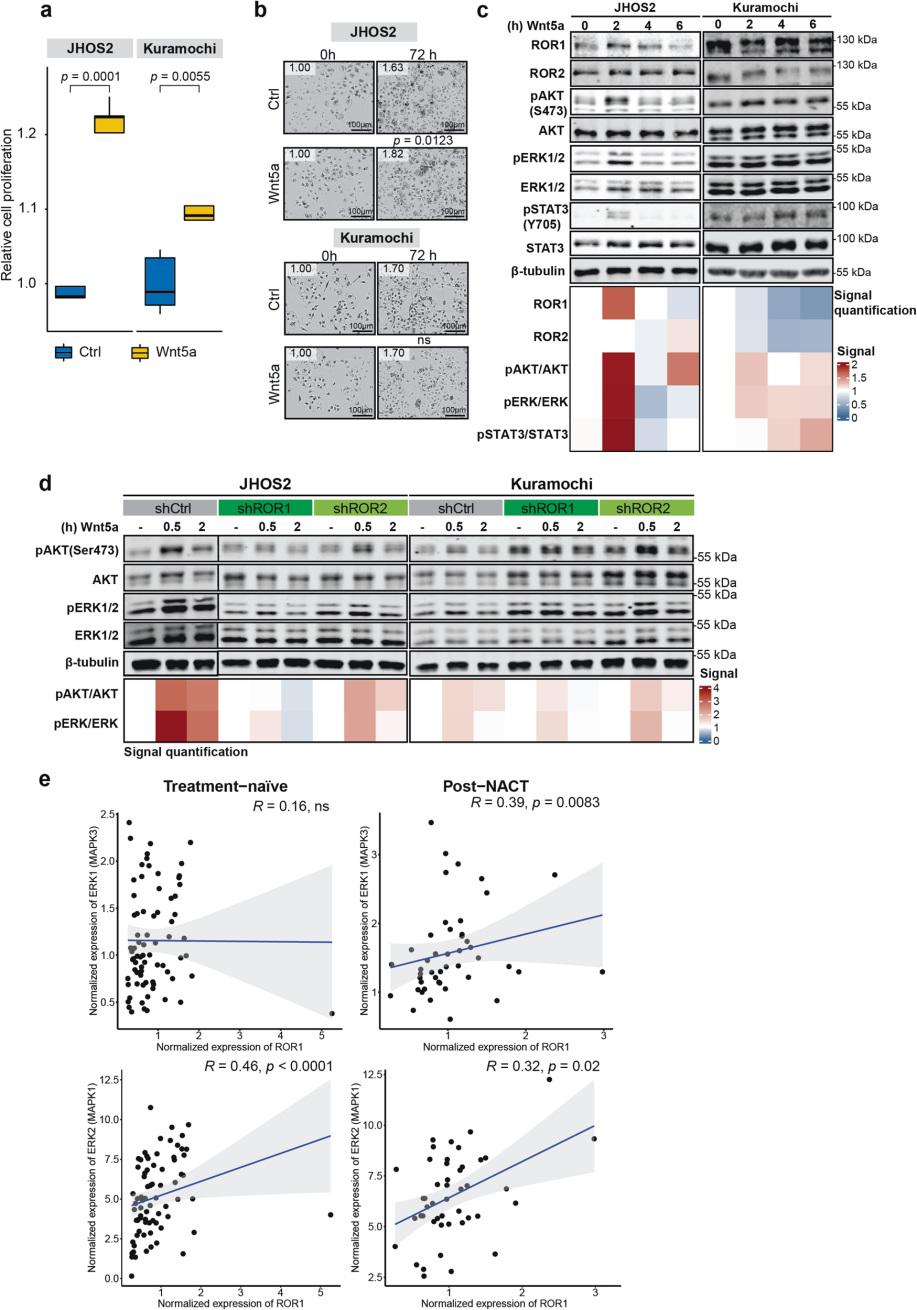
Wnt5a can activate pAKT/pERK/pSTAT3 in OC cells via binding to ROR1. **a** Boxplots reporting the relative proliferation measured via CTG of untreated (control sample) and Wnt5a treated (72 h, 100 ng/ml), serum starved JHOS2, and Kuramochi cells. The significance between the annotated samples was calculated with a one-tailed Welch *t*-test. **b** Representative microscopic images (Incucyte) of serum-starved JHOS2 and Kuramochi cells untreated (control samples) or treated with Wnt5a (72 h, 100 ng/ml). The depicted values represent the image-based relative proliferation compared to control samples, set as value 1. The significance of pairwise differences between confluence means in untreated vs. Wnt5a-treated cells was calculated using the one-tailed Welch *t*-test. ns, not significant. Scale bar = 100 µm. **c** Immunoblot analysis of serum-starved JHOS2 and Kuramochi cell lines stimulated (or not) with 100 ng/ml of Wnt5a for the indicated times. **d** Immunoblot analysis of DOX-treated (4 days) JHOS2 and Kuramochi shRNA cell lysates stimulated (or not) with 100 ng/ml of Wnt5a for indicated times. **e** Spearman correlation between the expression levels of ROR1 and ERK1/2 from the bulk RNA-seq data of the DECIDER clinical samples. The data refer only to the epithelial ovarian cancer cells. ns, not significant. For the signal quantification in (**c**) and (**d**), protein levels were normalized based on β-tubulin, which was used as a loading control, and then adjusted based on the unstimulated samples that were given value 1.

Wnt5a stimulation was able to mediate a modest increase in OC cell proliferation, more evident in JHOS2 compared to Kuramochi cells as measured by CTG (Cell Titer Glow) and Incucyte (Fig. 3a, b). Accordingly, these proliferative responses corresponded to a stronger pERK/pAKT/pSTAT3 activation in serum-starved and quiescent JHOS2 cells compared to Kuramochi cells (Fig. 3c). This difference could likely be due to higher basal activation of the AKT/ERK pathway related to a KRAS amplification in Kuramochi cells [31].

As both ROR1 and ROR2 receptors are Wnt5a binders, we wanted to evaluate the relative contribution of each receptor to Wnt5a-mediated pERK/pAKT activation in OC cells. Knockdown of ROR1 correlated with a marked loss of Wnt5a-mediated pERK/pAKT activation in JHOS2 cells, whereas loss of ROR2 elicited only a moderate effect (Fig. 3d). In quiescent Kuramochi cells, this effect was less intense, but a total loss in pAKT activation after 2 h of Wnt5a stimulation was observed in shROR1 cells (Fig. 3d). Of note, Wnt5a-mediated STAT3 phosphorylation could not be evaluated in this context due to the loss of STAT3 expression in shROR1 isogenic cells. Consistent with these in vitro assays, a positive correlation between ROR1–ERK1 (MAPK3) and ROR1–ERK2 (MAPK1) mRNA levels was observed in DECIDER post-NACT clinical samples, which earlier showed an elevated ROR1 expression (Spearman *r* = 0.39*, p* = 0.0083; Spearman *r* = 0.32, *p* = 0.02, respectively, Fig. 3e). On the other hand, we observed a negative correlation between ROR2–ERK1 (MAPK3) and ROR2–ERK2 (MAPK1) mRNA in both treatment-naïve and post-NACT samples (Suppl. Fig. 3). Taken together, our results show that the Wnt5a ligand can activate pERK/pAKT/pSTAT3 in OC cells via ROR1/ROR2 engagement, where ROR1 is more potent than ROR2 in activating the pERK/pAKT pathway.

### ROR1 expression is strongly detected in OC stromal cells such as CAFs

**Fig. 4:**
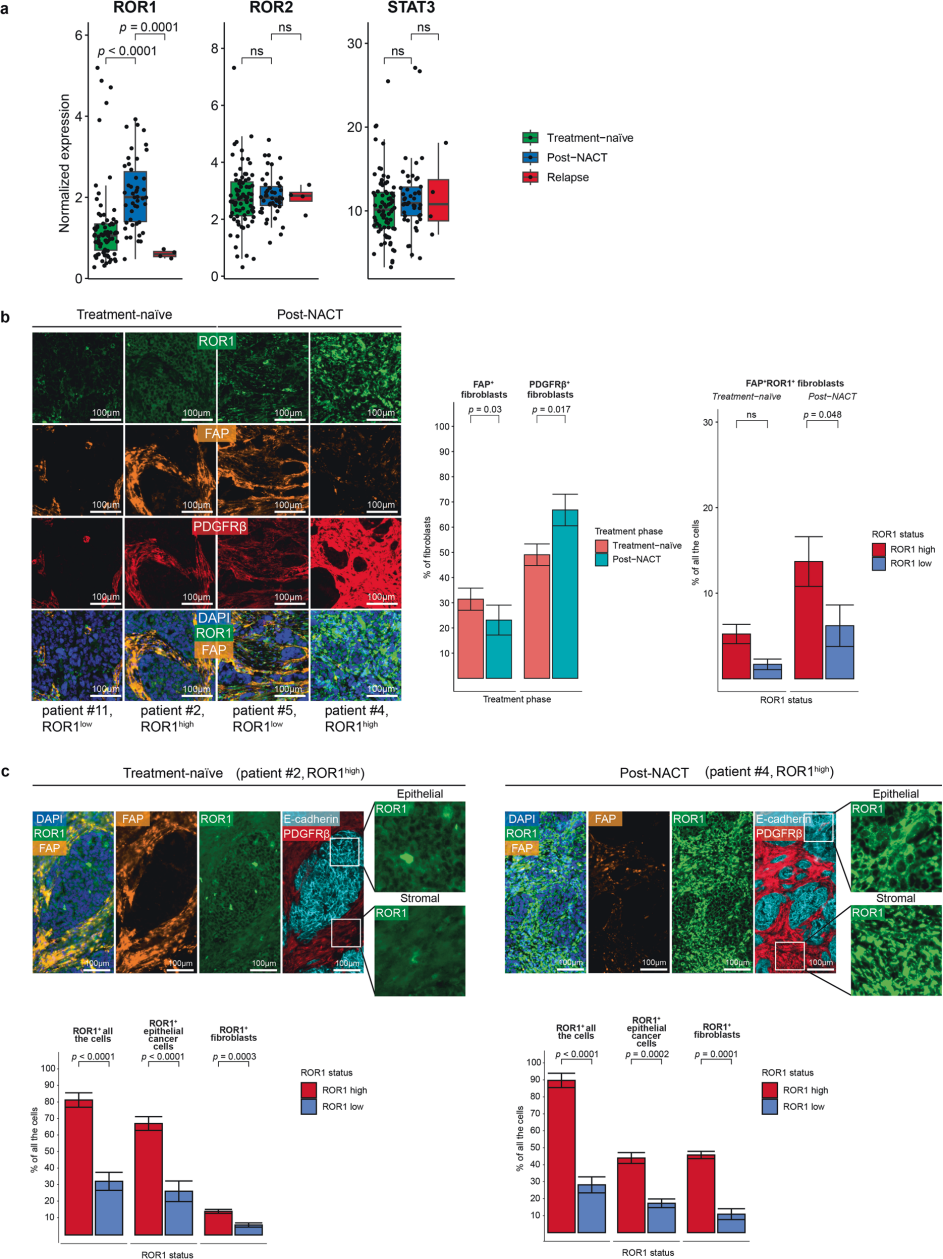
ROR1 is strongly expressed in OC fibroblasts, including CAFs. **a** Boxplots depicting the expression of ROR1, ROR2, and STAT3 in fibroblasts in treatment-naïve (*n* = 75), post-NACT (*n* = 46), and relapsed (*n* = 4) HGSOC patient samples in bulk RNA-seq data of the DECIDER cohort. **b** *Left:* representative mIHC images showing ROR1, FAP, and PDGFRβ expression in two treatment-naïve (patients #11 and #2) and two post-NACT (patients #5 and #4) patient samples. DAPI (in blue) was used for nuclear staining. The mIHC-based ROR1 classification is reported under the sample names. Scale bar = 100 µm. *Center and right:* mIHC data quantification shown as percentages of FAP^+^ or PDGFRβ^+^ fibroblasts normalized to the total number of fibroblasts (center) or percentages of FAP^+^ROR1^+^ fibroblasts normalized to the total cell count (right) in treatment-naïve and post-NACT samples with ROR1^high^ or ROR1^low^ classification. **c** *Top:* representative examples showing ROR1, FAP, E-cadherin, and PDGFRβ expression in one treatment-naïve and one post-NACT tumor sample. DAPI (in blue) was used as nuclear staining. White boxes show the area magnified on the right to emphasize ROR1 staining. The classification of patient samples based on ROR1 expression is reported above images. Scale bar = 100 µm. *Bottom*: mIHC data quantification shown as percentages of ROR1^+^ for all cells, ROR1^+^ for epithelial cancer cells, and ROR1^+^ for fibroblasts normalized to the total cell count in ROR1^high^ and ROR1^low^ samples within the two treatment phases. In **a**–**c**, the significance of the pairwise differences between means was calculated using the Wilcoxon rank-sum test. In **b**, **c** charts, the quantification is presented in the form of mean ± standard deviation. ns not significant.

To understand whether ROR1 expression is exclusive to cancer cells, we analyzed the bulk RNA sequencing (RNA-seq) data following sample decomposition of the DECIDER cohort into epithelial ovarian cancer cells and stromal cells [32]. Interestingly, we obtained high expressions of ROR1, ROR2, and STAT3 in fibroblasts (Fig. 4a), which was more obvious when compared to epithelial cancer cells across all treatment groups (Suppl. Fig. 4). This suggests that the non-canonical Wnt signaling is also strongly active in tumor-associated stromal cells. Therapeutically significant, post-NACT clinical samples were more enriched in fibroblasts compared to treatment-naïve samples (Suppl. Fig. 1), indicating that chemotherapy modulates OC tumor heterogeneity by increasing the stromal cell population.

To validate these findings, we performed mIHC analysis of FFPE (formalin-fixed paraffin-embedded) tissues from our retrospective OC cohort (*n* = 11) using a cocktail of antibodies to define specific cell types: PDGFRβ, fibroblast marker; FAP, cancer-associated fibroblasts (CAFs) marker; E-cadherin, epithelial cancer cell marker, combined with ROR1 and phospho-STAT3 (pSTAT3) Tyr705 staining. Histologically, the distribution of fibroblasts (or stromal cells) in the tumors varied, and we observed that, in general, post-NACT tumors displayed more regions with fibroblasts infiltrations (PDGFRβ^+^) among cancer cell areas than in treatment-naïve samples, which were more homogenous (Fig. 4b, Suppl. Fig. 4b). From each FFPE slide, six regions of interest (ROIs) were chosen across all tissue sections for mIHC analysis (Suppl. Fig. 4c, d). For a better characterization of ROR1^+^ cells, we classified the patient samples as ROR1^low^ or ROR1^high^ based on the average ROR1 expression in all six ROIs. We detected ROR1 expression in both PDGFRβ^+^/FAP^+^ fibroblasts, and accordingly, post-NACT samples showed a higher percentage of FAP^+^ROR1^+^ cells with ROR1^high^ expression than treatment-naïve samples (Fig. 4b). Moreover, the percentage of ROR1^high^ fibroblasts were considerably higher in post-NACT samples (50%) compared to treatment-naïve samples (20%, Fig. 4c, Suppl. Fig. 4e). Taken together, our mIHC analysis showed that post-NACT samples are more enriched in fibroblasts than treatment-naïve samples, and most of these fibroblasts are also high in ROR1 expression.

### High ROR1/STAT3 expression is detected in stromal cells and defines a pro-inflammatory transcriptomic signature in OC

**Fig. 5:**
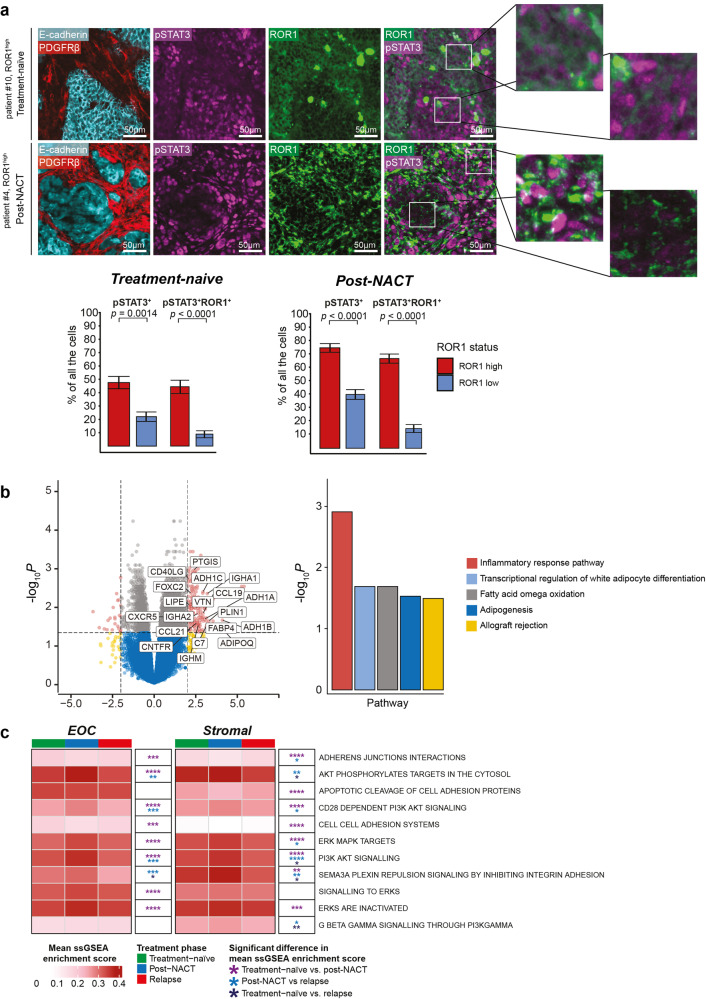
ROR1 and STAT3 define a common gene-expression signature in OC. **a** *Top:* representative examples showing E-cadherin and PDGFRβ staining and co-localization of pSTAT3 Y705 and ROR1. White boxes indicate the magnified area. Scale bar = 50 µm. *Bottom:* percentages of pSTAT3^+^ and pSTAT3^+^ROR1^+^ cells normalized to the total cell count in treatment-naïve and post-NACT samples. The quantification reported in the charts is presented in the form of mean ± standard deviation, and the significance of the differences among means was calculated with the Wilcoxon rank-sum test. **b** *Left:* volcano plot depicting the statistical significance and magnitude of change in mRNA expression levels between STAT3^high^ & ROR1^high^ (*n* = 21) vs. STAT3^low^ & ROR1^low^ (*n* = 21) samples. The labeled genes (*P* < 0.05, log_2_FC > 2) in STAT3^high^ & ROR1^high^ are enriched for five pathways reported in the bar chart (right) and associated with inflammation and adipogenesis. The expression data refers only to the epithelial ovarian cancer cells. c. Heatmap reporting the activation status of eleven REACTOME pathways related to PI3K, AKT, ERK, adhesion, and junctions. The activation status was quantified as ssGSEA (single sample gene set enrichment analysis) mean enrichment score for each RNA-seq-deconvoluted cell type (ovarian cancer epithelium or stroma) and treatment phase. The wildcard indicates the significance of the difference between the two treatment groups, evaluated via the Wilcoxon rank-sum test (*P ≤ 0.05; **P ≤ 0.01; ***P ≤ 0.001; ****P ≤ 0.0001). The color of the wildcard characters refers to the comparison of references.

Since we identified STAT3 as a downstream transcription factor modulated by ROR1 expression, we also investigated whether ROR1^+^ tumor cells are pSTAT3^+^ (Y705). Accordingly, we detected pSTAT3 staining in both fibroblasts (PDGFRβ^+^) and cancer cells (E-cadherin^+^), with a slightly bigger proportion of pSTAT3^+^ cells in post-NACT samples (Fig. 5a). Noteworthy, we identified ROR1 and pSTAT3 staining in the same tumor cells (Fig. 5a), which indicates that these proteins are co-expressed and could modulate OC tumor heterogeneity. ROR1 expression could be detected in some cells in the absence of pSTAT3, but pSTAT3 expression seemed to be, to a large extent, co-localized with ROR1^+^ cells. Moreover, a positive correlation between pSTAT3^low^/ROR1^low^ or pSTAT3^high^/ROR1^high^ staining was observed in both treatment-naïve and post-NACT samples for all cells, with some variations among epithelial cancer cells and stromal cells (Suppl. Fig. 5a–l). The strongest correlation for pSTAT3^high^/ROR1^high^ staining was observed in treatment-naïve cancer cells (Suppl. Fig. 5g, Spearman *r* = 0.47*, p* = 0.0007) and post-NACT stromal cells (Suppl. Fig. 5i, Spearman *r* = 0.7*, p* = 0.0011), which is in line with our previous observation that post-NACT samples have more stromal cells.

To unveil the biological processes mediated by ROR1 and STAT3 signaling in OC, we stratified the DECIDER samples according to their ROR1 and STAT3 gene expression levels. Specifically, the samples whose ROR1 mRNA expression was strictly greater or smaller than the median were appointed as ROR1^high^ or ROR1^low^ samples, respectively, and a similar approach was adopted to identify STAT3^high^ and STAT3^low^ samples. Then, a differential gene expression analysis was performed between ROR1^high^ & STAT3^high^ and ROR1^low^ & STAT3^low^ (*n* = 21) expression groups (Suppl. Table 3). We uncovered several genes significantly upregulated in ROR1^high^ and STAT3^high^ samples. Among those, we found several molecules involved in adipogenesis and lipid-associated signaling modulation, such as aldehyde dehydrogenase 1 (ADH1) gene family members A–C; adiponectin (ADIPOQ), lipase E (LIPE) and perilipin-1 (PLIN1, Fig. 5b, Suppl. Table 3). Moreover, several inflammatory-related chemokines such as C-X-C motif chemokine receptor 5 (CXCR5), C-C motif chemokine ligand 19 (CCL19), and 21 (CCL21) were also overexpressed in ROR1^high^ & STAT3^high^ samples. Accordingly, functional enrichment analyses of the highly upregulated genes in the ROR1^high^ & STAT3^high^ group (*p* < 0.05, log_2_FC > 2) unveiled the inflammatory response pathway and several adipogenesis-related functions as the main molecular processes associated with ROR1 and STAT3 signaling in these OC tumors (Fig. 5b, Suppl. Table 4a, b).

As NACT was able to modulate ROR1 expression at both the RNA and protein levels, we focused our analysis on the global effects of chemotherapy on ROR1-associated signaling pathways in OC, such as PI3K/AKT/ERK. To this aim, we proceeded with ssGSEA (single sample gene set enrichment analysis [33]) to computationally derive the divergences and similarities in the signaling profile of OC samples at different treatment phases and in both cancer and stromal cells. (Suppl. Tables 5a, b). As a result, NACT was shown to alter the activation status of PI3K/AKT and ERK/MAPK in both cell subtypes. In particular, the hyperactivity of such pathways observed in post-NACT cancer cells and fibroblasts was followed by a significant decrease of their scores in relapsed samples (Fig. 5c, Suppl. Table 5c). On the other hand, cell-type-specific signaling activity was also noticeable. For instance, adhesion-like processes were more enriched in, and thus more specific to, epithelial ovarian cancer cells compared to the stroma, whereas GPCRs-related signaling, such as G:βγ signaling through PI3Kγ emerged as distinctively overactive in fibroblasts. (Fig. 5c, Suppl. Table 5c).

## Discussion

Our studies show that the non-canonical Wnt signaling via ROR1/2 receptors can regulate phenotypic outputs in OC, contributing to tumor heterogeneity and disease progression. Previous studies have underlined the functional importance of ROR1/2 expression in OC, indicating that both receptors contribute to disease progression and chemoresistance [11]. In particular, ROR1 expression has been found in less-differentiated, aggressive HGSOC tumors and linked to shorter DFS or OS of OC patients [16]. Moreover, OC tumors with high ROR1 levels were enriched for the expression signature of genes associated with cancer stem cells and EMT, which facilitates cancer cell migration, metastatic development, and drug resistance [8]. ROR2 expression has also been found in primary and metastatic OC tumors and linked to cancer cell adhesion, proliferation, EMT, and chemoresistance [17, 34]. Our RNA-seq analysis of HGSOC tumor samples identified increased ROR1 expression in post-NACT samples, consistent with previous findings on the therapeutic modulation of ROR1, notably by glucocorticoid-enriched NACT that could induce ROR1 expression in OC cells and a ROR1-dependent metastatic development in breast cancer, respectively [18, 24]. Overall, the transcriptomic levels of ROR1 were higher than ROR2 in epithelial cancer cells from both treatment naïve and post-NACT HGSOC clinical samples (Fig. 1a). Furthermore, we observed that NACT mediated ROR1 transcriptional upregulation in both cancer cells and fibroblasts, and this data was corroborated by mIHC analysis (Fig. 4a, b; Suppl. Fig. 4a). Consequently, we could postulate that high ROR1 expression could contribute to high stromal content. It remains to be seen how ROR1-signaling activation in stromal cells could sustain the enrichment of these cells in post-NACT samples. Functionally, we showed that Wnt5a-binding to ROR1 could activate the pERK/pAKT pathway in ROR1^+^ OC cell lines and unveiled a positive correlation between ROR1 and MAPK1 (or ERK2) in both treatment-naïve and post-NACT samples. Altogether, our findings underscore ROR1 as a driver of ERK/AKT oncogenic signaling, as previously suggested [20]. On the other hand, the knockdown of ROR2 slightly attenuated Wnt5a-mediated pERK/pAKT activation, which would indicate that ROR2 could work to enhance the ROR1 signalosome, possibly as a ROR1 dimerization partner.

Furthermore, we identified STAT3 as a downstream ROR1 target in OC cells, which is in line with previous findings in ROR1^+^ leukemic cells [26] and gastric cancer [28]. Aberrant STAT3 activation has previously been linked with OC progression by mediating cell proliferation, survival, invasion, stemness, and drug resistance [35]. More importantly, constitutively phosphorylated STAT3 (Tyr705) level was found in patient ascites and ascites-derived OC cells [36]. A transcriptional-based feedback loop regulating STAT3 and ROR1 expression has been proposed, which suggests that IL-6-induced STAT3 binds to the ROR1 promoter to activate its transcription [37]. Our results showed that targeting ROR1 resulted in STAT3 downregulation in OC cells, indicating that ROR1 could also regulate STAT3 levels. This mechanism was investigated by Chen et al., showing that Wnt5a binding to ROR1 enhanced pSTAT3 Y705 levels in chronic lymphocytic leukemia (CLL), and this event was ROR1 dependent as in cells lacking ROR1 or treated with ROR1 mAb cirmtuzumab, Wnt5a-mediated pSTAT3 was not observed [26]. Interestingly, high IL-6 levels were secreted by ROR1-expressing CLL cells following Wnt5a stimulation, resulting in high pSTAT3 Y705 in these cells.

We found a strong pSTAT3 staining in our mIHC analysis, which was detected in both cancer cells (E-cadherin^+^) and stromal cells (PDGFRβ^+^), including CAFs (FAP^+^). A high level of pSTAT3 by means of Y705 phosphorylation would indicate that the pro-inflammatory cytokines such as IL-6, IL-8, and IL-11, together with many chemokines and modulators, are actively stimulating these cells [38, 39]. The presence of ROR1 and pSTAT3 in both epithelial cancer cells and stromal cells clearly indicates that ROR1/STAT3 signaling sustains the OC tumor microenvironment. This data was corroborated by our transcriptomics analysis of samples obtained from post-NACT samples of the DECIDER trial, revealing higher ROR1, ROR2, and STAT3 levels in OC stromal cells compared to epithelial cancer cells (Suppl. Fig. 4a). Interestingly, the expression of ROR1 in OC stromal cells was previously observed in a pan-cancer IHC analysis of OC subtypes [13]. However, the identity of the stromal cells was not defined.

Assuming that pSTAT3 is an indicator of pro-inflammatory processes in OC tumors, FAP^+^ stromal cells could represent the inflammatory subtype of CAFs. Previous studies of cell heterogeneity in OC by single-cell RNA-seq (scRNA-seq) analysis have documented the presence of inflammatory CAFs in the primary tumors and ascites samples of HGSOC patients, whereas hyperactivated JAK/STAT signaling was also prevalent in cancer cells [40]. In line with this, an early study has shown an increase in pSTAT3 levels in the recurrent metastatic lesions compared with the primary metastasis of OC patients, and a positive correlation between pSTAT3 expression and the presence of intra-tumoral inflammatory cell infiltration was found [41]. Interestingly, our differential gene expression analysis of samples from patients with HGSOC based on ROR1^high^ & STAT3^high^ expression status revealed enrichment in adipogenesis-related biological functions, which suggests a gene expression signature closely resembling the omentum’s transcriptional fingerprint. The omentum is a preferred metastatic site for OC cells, and the majority of HGSOC patients at advanced stage have omental metastases [42]. Resident immune cells in the omentum provide a niche for metastatic cells to grow via the upregulation of the JAK/STAT pathway, and in particular, through STAT3 hyperactivity [43]. Wnt5a expression was also enriched in OC samples [44] and was found in the OC tumor microenvironment of visceral adipose cells [45], whereas ROR1 expression was detected in human adipose cells [19].

Our transcriptomics and mIHC analysis demonstrated that post-NACT samples are more enriched in stromal cells compared to treatment-naïve samples. Stromal cells are known to modulate the extracellular matrix (ECM) architecture and contribute to therapy resistance [46] via various mechanisms. Therefore, a stroma-enriched post-NACT OC tumor could be more challenging to treat. Taken together, it is conceivable that Wnt5a-ROR1/STAT3 signaling in OC samples reflects a phenotype associated with pro-inflammatory traits for both epithelial ovarian cancer and stromal cells through a feedback loop that involves a STAT3-mediated upregulation of Wnt5a/ROR1 signaling, which in turn could initiate STAT3 signaling via pro-inflammatory pathways (Suppl. Fig. 6). It will be of high interest to investigate the phenotypic profile of omentum-derived OC metastases in terms of Wnt5a-ROR1/STAT3 activation and interrogate the therapeutic potential of targeting ROR1 and JAK/STAT signaling to interfere with omental metastatic development.

## Materials and methods

### Ethics approval

The study and the use of all clinical material for RNA sequencing have been approved by The Ethics Committee of the Hospital District of Southwest Finland (ETMK) under decision number EMTK: 145/1801/2015. Patient material for IHC and mIHC was obtained from Tampere University Hospital. The use of all the clinical material has been approved by The Regional Ethics Committee of the Expert Responsibility area of Tampere University Hospital under decision number: R09108/R11137.

More information on Materials and Methods is available in Supplementary Materials and Methods, and whole blots of the immunoblots shown in the main figures are shown in Suppl. Fig. 7. Antibody details are reported in Suppl. Table 6.

## Supplementary information

Supplementary Materials and Methods

Original Data Files

Supplementary Figure 1

Supplementary Figure 2

Supplementary Figure 3

Supplementary Figure 4

Supplementary Figure 5

Supplementary Figure 6

Supplementary Table 1

Supplementary Table 2

Supplementary Table 3

Supplementary Table 4

Supplementary Table 5

Supplementary Table 6

Supplementary Table 7

## Data Availability

All data relevant to the study are included in the article or uploaded as supplementary information and are available on reasonable request from the corresponding author.

## Code availability

All codes relevant to the study are available on reasonable request from the corresponding author.
