# Supplementary material for: ROR1-STAT3 signaling contributes to ovarian cancer intra-tumor heterogeneity
Source: Cell Death Discov. 2023 Jul 3;9:222. doi: 10.1038/s41420-023-01527-6 (PMC10317980; doi:10.1038/s41420-023-01527-6)
Supplement: Supplementary file 1 — Supplementary Materials and Methods [file 41420_2023_1527_MOESM1_ESM.docx]

# **Supplementary Material and Methods**

## **Cell culture**

Ovarian cancer cell line Kuramochi was obtained from JCBR cell bank and grown in RPMI 1640 media (Lonza, Basel, Switzerland) supplemented with 10% fetal bovine serum, 2 mM L-glutamine, and Primocin™ (InvivoGen, San Diego, CA, USA). JHOS2 cells were obtained from Riken BRC and grown in DMEM/F-12 media (Gibco™, Thermo Fisher Scientific™, Waltham, MA, USA) supplemented with 10% fetal bovine serum, 1X MEM non-essential amino acid solution (Thermo Fisher Scientific™, Waltham, MA, USA) and Primocin™. Cell counting was done with Countess II automated cell counter (Invitrogen™, Thermo Fisher Scientific™). Stable shRNA cell lines expressing control-, ROR1- and ROR2-shRNA were generated as described previously [1,2] by using an inducible pLKO-Tet-On lentiviral expression system. Expression of shRNA was induced by 100 ng/ml doxycycline (DOX) treatment for at least three days and ROR1/ROR2 silencing was verified by immunoblot analysis.

## **Cell proliferation assay and Wnt stimulation**

Prior stimulation, cells were serum starved for 24 h, after which Wnt5a (645-WN; R&D Systems, Minneapolis, MN, 100 ng/ml) was added. Relative cell proliferation was measured in two different ways, by CellTiter-Glo (CTG) assay and IncuCyte S3 Live-Cell Analysis System (Sartorius, Göttingen, Germany). CTG 2.0 Assay (Promega, Madison, WI, USA) was used to measure cell viability according to manufacturer’s instructions. Luminescence signal was detected using Envision plate reader (PerkinElmer, Waltham, MA, USA) or PHERAstar FS (BMG Labtech, Ortenberg, Germany). For the IncuCyte assay, the cells were seeded in 96-well plates, 1000 cells in 100 µl media per well. Five replicates were used per condition. After 24 h starvation, Wnt5a was added to selected wells at 100ng/ml. Automated microscopic images (5X objective, phase contrast) were taken at timepoints 0 h and 72 h, and confluences were determined by Incucyte S3 image analysis software.

## **Immunoblotting**

Cells were lysed with Triton-X lysis buffer (50 mM Tris-HCl pH 7.5, 10% glycerol, 150 mM NaCl, 1 mM EDTA, 1% Triton-x-100, 50 mM NaF) supplemented with protease and phosphatase inhibitor cocktails (Pierce Protease and Phosphatase Inhibitor Mini Tablets, Thermo Scientific™, MA, USA). Lysates were mixed with Laemmli sample buffer (Bio-Rad Laboratories, California, USA), separated in SDS-PAGE, and transferred to nitrocellulose membranes. The primary antibodies used for immunoblotting are listed in Suppl. Table 6. Secondary antibodies were IRDye® 800CW Donkey anti-Mouse IgG or IRDye® 680RD Donkey anti-Rabbit IgG (LI-COR, Lincoln, NE, USA) and blots were scanned with Odyssey® Fc Imaging system (LI-COR). Image analysis was done using the Image Studio Lite software (LI-COR). The whole blots for the immunoblots shown in the main figures are shown in Suppl. Fig. 7.

## **Proteomic analysis**

For each cell line (Kuramochi and JHOS2) and for each condition (shCtrl, shROR1, shROR2), three biological replicates underwent proteomics analyses via liquid chromatography-tandem mass spectrometry (LC-MS/MS). Protein concentrations were measured from thawed cell lysates using a BCA protein assay kit (Thermo Scientific™). 300 µg of protein was then taken from each sample, and sample volumes were adjusted to 300 µl 8M urea on ice. Cysteine bonds were reduced with 5 mM Tris(2-carboxyethyl) phosphine (TCEP) for 30 min at +37 °C, followed by alkylation with 10 mM iodoacetamide for 20 min in the dark. 1 ml of 50 mM AMBIC was then added to neutralize the urea buffer. Proteins were digested to peptides with sequencing grade modified trypsin (Promega V5113), at 1:100 enzyme:protein ratio at +37 °C overnight. The following day, pH of the samples was adjusted to < 3 with TFA, and ACN was added to a final concentration of 1% (v/v). Samples were desalted with BioPureSPN Macro Desalting columns. The columns were conditioned with 200 µl of ACN and centrifuged for 1 min at 55 g, followed by flushing with 200 µl of MilliQ water and 1 min 55 g centrifugation. Column was then equilibrated with 200 µl of buffer A (0.1% TFA, 1% ACN), after which the samples were processed 200 µl at a time, followed by two washes with buffer A. Finally, samples were released with 200 µl of elution buffer (80% ACN, 0.1% TFA). The elution step was repeated three times, after which the samples were dried in a vacuum centrifuge. Liquid chromatography-tandem mass spectrometry (LC-MS/MS) was performed, and the spectra acquired as described in [3]. For the total proteome samples, three biological replicates were used, and for each run, 2 µl of sample was injected. The total number of proteins identified per sample before and after filtering is available in Suppl. Table 7. The proteomics statistical analyses were performed as in [4] using R (version 4.2.1 [5]) and the DEP R package (version 1.16.0 [6]).

## **Bulk RNA sequencing (RNA-seq) of clinical samples**

The sample collection and analysis are part of the DECIDER cohort (<https://www.deciderproject.eu/> [7]). The sample cohort consists of 82 patients that were treated for high grade serous ovarian cancer (HGSC) at Turku University Hospital between November 2011 and August 2021 (diagnose time: November 2011 to October 2019). From the cohort, 125 solid intra-abdominal peritoneal or omental metastatic samples were collected. Of these, 75 originated from treatment-naïve, 46 from post-NACT, and four came from relapsed tumors. Bulk RNA-seq reads were preprocessed using the SePIA pipeline [8] within the Anduril framework [9]. Read pairs were trimmed using Trimmomatic (version 0.33 [10]) and aligned to the GRCh38.d1.vd1 reference genome with GENCODE v25 annotation using STAR (version 2.5.2b [11]). Gene level effective counts (found to be more accurate than the raw read counts) were quantified using eXpress (version 1.5.1-linux_x86_64 [12]). The batch effects between different library preparation protocols were removed using POIBM (<https://bitbucket.org/anthakki/poibm/> [13]).

The epithelial ovarian cancer cell-, fibroblast- and immune-specific expressions were estimated using PRISM [14], and these levels were used for downstream analysis with R [5]. Treatment-naïve samples reporting a normalized expression level of ROR1 or STAT3 strictly greater/smaller than or equal to their median across all the samples were categorized as ROR1^high^/ROR1^low^ and STAT3^high^/STAT3^low^, respectively. Then, the intersection of the ROR1^high^ and STAT3^high^ as well as ROR1^low^ and STAT3^low^ was taken, identifying the “high” and “low” expression samples, each including 21 samples. Lastly, differential expression analysis between these two groups was run using the R package limma (version 3.52.4 [15,16]). Functional enrichment analysis was performed using GeneTrail2 (over-representation analysis, *Homo Sapiens,* all the categories) [17], focusing on the top five significant results from ConsensusPathDB’s WikiPathways (<https://www.wikipathways.org/> [18]). The cancer cell and fibroblasts’ deconvoluted data underwent single sample gene set enrichment analysis (ssGSEA [19]) via the R package GSVA (version 1.44.5 [20]) and using the 430 REACTOME pathways (<http://www.reactome.org>) in the C2 collection of Molecular Signatures Database (MSigDB) 3.0 Gene Sets (<https://www.gsea-msigdb.org/gsea/msigdb>). For the ssGSEA mean enrichment scores’ comparisons, pertinent pathways containing the words STAT3|PI3K|AKT|ADHESION|JUNCTIONS|ERK were selected, and the significance of the Wilcoxon rank-sum test results was calculated with the R package sigminer (version 2.1.9 [21]). For both the differential expression analyses and the ssGSEA Wilcoxon rank-sum tests, the p-values were adjusted according to Benjamini and Hochberg [22].

## **Immunohistochemistry (IHC)**

Patient material for IHC and mIHC was obtained from Tampere University Hospital as ready sectioned slides from Formalin-Fixed Paraffin-Embedded (FFPE) -blocks. The sectioned tumor tissue slides were deparaffinized in xylene (three 5 min incubations) and rehydrated in decreasing ethanol series (99,8%, 96%, 70%; 2 min incubations), followed by heat-induced epitope retrieval (HIER) in 10 mM Tris-HCl – 1 mM EDTA buffer (pH 9) at +99 °C for 20 min (PT Module, Thermo Scientific™, Waltham, MA, USA) and washing slides 5 min in MilliQ water and Tris-buffered saline (TBS). Endogenous peroxidase activity was blocked with incubation in 0.9% H_2_O_2_ in TBS for 15 min, followed by three of 3 min TBST (TBS - 0,05% Tween20) washes and blocking with 10% normal goat serum (Gibco™, Thermo Fisher Scientific™, Waltham, MA, USA) in TBST for 15 min.

For the staining, primary antibodies (PAX8 and ROR1, antibody details Suppl. Table 6) were diluted to 10% normal goat serum in TBST and applied to the slides. The slides were incubated for 90 min at room temperature (RT) in the humid chamber, followed by three TBST washes (3 min each). Secondary antibodies (Immunologic, Netherlands) were applied and incubated for 30 min in RT followed by TBST washes. 3,3’-diaminobenzidine (BrightDAB, Immunologic, Netherlands) solution was applied to the slides for 5 min followed by 1 min wash in MilliQ water. Counter staining was done through 1 min incubation in 1:10 diluted Mayer’s hematoxylin (Dako, CA, USA) followed by a 5 min water wash. Dehydration was performed in increasing ethanol series (70%, 96%, 99,8%; 1 min incubations) and xylene (three of 1 min incubations), after which coverslips were inserted prior to imaging of the slides. ROR1 intensity in IHC staining was evaluated on PAX8 positive areas and scored 0-3 (Suppl. Fig. 8).

## **Multiplex immunohistochemistry (mIHC)**

The primary antibody panel included five antibodies: ROR1, FAP, pSTAT3, PDGFRβ and E-cadherin, and two rounds of staining and scanning were performed (antibody panel details Suppl. Table 6). The antibody panel was modified from the earlier described fibroblast panel [23] and mIHC staining and image analysis protocols were modified from [24].

*First round of staining*. The tissue was prepared as described in the IHC section. Primary antibodies were diluted in 10% normal goat serum (in TBST), applied to the slides and incubated overnight at +4 °C. Slides were then washed three times (3 min) in TBST, and the secondary antibodies (anti-mouse or anti-rabbit horseradish peroxidase (HRP)-conjugated (Immunologic, Netherlands)), diluted 1:5 to TBST, were applied for 30 min, followed by TBST washes. Tyramide signal amplification (TSA (PerkinElmer, Waltham, MA, USA) 1:100 in TBST, 1,5‰ H_2_O_2_) was applied for 15 min followed by TBST washes and peroxidase block as described in the IHC section. The second TSA reaction was performed as described above, followed with heat-induced epitope retrieval in 20 mM Tris-HCl – 1 mM EDTA buffer (pH 9).

After normal goat serum blocking, primary antibodies were applied and incubated overnight as described above. Secondary antibodies (AlexaFluor647 or AlexaFluor750 together with DAPI (Thermo Fisher Scientific™), were diluted 1:300 to TBST and applied for 30 min at RT, followed by TBST washes, drying the slides and mounting the coverslips with ProLong Gold (Thermo Fisher Scientific™, Waltham, MA, USA). Fluorescence was scanned using appropriated filters of AxioImager.Z1 (Zeiss, Germany).

*Second round of staining.* The coverslips were detached with TBST incubation overnight at +4 °C. To remove previous AlexaFluor-staining, slides were immersed in bleaching solution, 4,5% H2O2 + 24mM NaOH in TBS, under a white light lamp for 1 h. TBST washes, HIER and normal goat serum blocking were performed as described above, followed by the next round of AlexaFluor -staining and a second scanning round.

## **Image analysis of mIHC data**

The staining quality was visually evaluated in the whole tissues and six 1 mm x 1 mm representative regions of interest (ROIs) per sample were chosen and cropped for image analysis, avoiding the edges of tissue and folded tissue parts (Suppl. Fig. 3b). ROIs were exported from ZEN Blue (Zeiss, version 3.1) as 100% TIFFs. The first and the second scanning round channels were aligned based on DAPI staining using MATLAB (version 9.12.0, R2022a [25]), and nuclear segmentation was carried out with Python (version 3.9.7 [26]). RGB images for epithelial-stromal classification, red blood cell and autofluorescence masks were created with CellProfiler (Suppl. Fig. 3c, version 4.2.1 [27]). Machine-learning based masks were created with Ilastik (version 1.3.3 [28]), and the masks were exported for each ROI. The epithelial-stromal classification mask was created based on E-cadherin (epithelial cells) and PDGFRβ (stromal cells) staining and morphology. The thresholds for each antibody were determined and image analysis was performed with CellProfiler. The image analysis protocol was modified from [23].

1. Wiederschain, D. *et al.* Single-vector inducible lentiviral RNAi system for oncology target validation. *https://doi.org/10.4161/cc.8.3.7701* **8,** 498–504 (2009).

2. Karvonen, H., Niininen, W., Murumägi, A. & Ungureanu, D. Targeting ROR1 identifies new treatment strategies in hematological cancers. *Biochem. Soc. Trans.* **45,** 457–464 (2017).

3. Salokas, K. *et al.* Physical and functional interactome atlas of human receptor tyrosine kinases. *bioRxiv* 2021.09.17.460748 (2021). doi:10.1101/2021.09.17.460748

4. Raivola, J. *et al.* Multiomics characterization implicates PTK7 in ovarian cancer EMT and cell plasticity and offers strategies for therapeutic intervention. *Cell Death Dis. 2022 138* **13,** 1–12 (2022).

5. R: A language and environment for statistical computing. at https://www.r-project.org/. (2022)

6. Zhang, X. *et al.* Proteome-wide identification of ubiquitin interactions using UbIA-MS. *Nat. Protoc. 2018 133* **13,** 530–550 (2018).

7. Lahtinen, A. *et al.* Evolutionary states and trajectories characterized by distinct pathways stratify patients with ovarian high grade serous carcinoma. *Cancer Cell* (2023). doi:10.1016/J.CCELL.2023.04.017

8. Icay, K. *et al.* SePIA: RNA and small RNA sequence processing, integration, and analysis. *BioData Min.* **9,** (2016).

9. Cervera, A. *et al.* Anduril 2: upgraded large-scale data integration framework. *Bioinformatics* **35,** 3815–3817 (2019).

10. Bolger, A. M., Lohse, M. & Usadel, B. Trimmomatic: a flexible trimmer for Illumina sequence data. *Bioinformatics* **30,** 2114–2120 (2014).

11. Dobin, A. *et al.* STAR: ultrafast universal RNA-seq aligner. *Bioinformatics* **29,** 15–21 (2013).

12. Roberts, A. & Pachter, L. Streaming fragment assignment for real-time analysis of sequencing experiments. *Nat. Methods 2012 101* **10,** 71–73 (2013).

13. Holmström, S., Hautaniemi, S. & Häkkinen, A. POIBM: batch correction of heterogeneous RNA-seq datasets through latent sample matching. *Bioinformatics* **38,** 2474–2480 (2022).

14. Häkkinen, A. *et al.* PRISM: recovering cell-type-specific expression profiles from individual composite RNA-seq samples. *Bioinformatics* **37,** 2882–2888 (2021).

15. Ritchie, M. E. *et al.* limma powers differential expression analyses for RNA-sequencing and microarray studies. *Nucleic Acids Res.* **43,** e47 (2015).

16. Law, C. W., Chen, Y., Shi, W. & Smyth, G. K. Voom: Precision weights unlock linear model analysis tools for RNA-seq read counts. *Genome Biol.* **15,** 1–17 (2014).

17. Stöckel, D. *et al.* Multi-omics enrichment analysis using the GeneTrail2 web service. *Bioinformatics* **32,** 1502–1508 (2016).

18. Herwig, R., Hardt, C., Lienhard, M. & Kamburov, A. Analyzing and interpreting genome data at the network level with ConsensusPathDB. *Nat. Protoc. 2016 1110* **11,** 1889–1907 (2016).

19. Barbie, D. A. *et al.* Systematic RNA interference reveals that oncogenic KRAS-driven cancers require TBK1. *Nat. 2009 4627269* **462,** 108–112 (2009).

20. Hänzelmann, S., Castelo, R. & Guinney, J. GSVA: Gene set variation analysis for microarray and RNA-Seq data. *BMC Bioinformatics* **14,** 1–15 (2013).

21. Wang, S. *et al.* Copy number signature analysis tool and its application in prostate cancer reveals distinct mutational processes and clinical outcomes. *PLOS Genet.* **17,** e1009557 (2021).

22. Benjamini, Y., Hochberg, Y. & Benjamini, Yoav, H. Y. Controlling the False Discovery Rate: A practical and Powerful Approach to Multiple Testing. *J. R. Stat. Soc. Ser. B* **57,** 289–300 at http://www.stat.purdue.edu/~doerge/BIOINFORM.D/FALL06/Benjamini and Y FDR.pdf%5Cnhttp://engr.case.edu/ray_soumya/mlrg/controlling_fdr_benjamini95.pdf (1995)

23. Pellinen, T. *et al.* Fibroblast subsets in non-small cell lung cancer: Associations with survival, mutations, and immune features. *JNCI J. Natl. Cancer Inst.* (2022). doi:10.1093/JNCI/DJAC178

24. Blom, S. *et al.* Systems pathology by multiplexed immunohistochemistry and whole-slide digital image analysis. *Sci. Reports 2017 71* **7,** 1–13 (2017).

25. The MathWorks Inc. MATLAB version: 9.12.0 (R2022a). at https://www.mathworks.com (2022)

26. van Rossum, G. Python reference manual. at (1995)

27. Stirling, D. R. *et al.* CellProfiler 4: improvements in speed, utility and usability. *BMC Bioinformatics* **22,** 1–11 (2021).

28. Berg, S. *et al.* ilastik: interactive machine learning for (bio)image analysis. *Nat. Methods 2019 1612* **16,** 1226–1232 (2019).
