# Supplementary material for: ROR1-STAT3 signaling contributes to ovarian cancer intra-tumor heterogeneity
Source: Cell Death Discov. 2023 Jul 3;9:222. doi: 10.1038/s41420-023-01527-6 (PMC10317980; doi:10.1038/s41420-023-01527-6)

Figure 2, uncropped WB

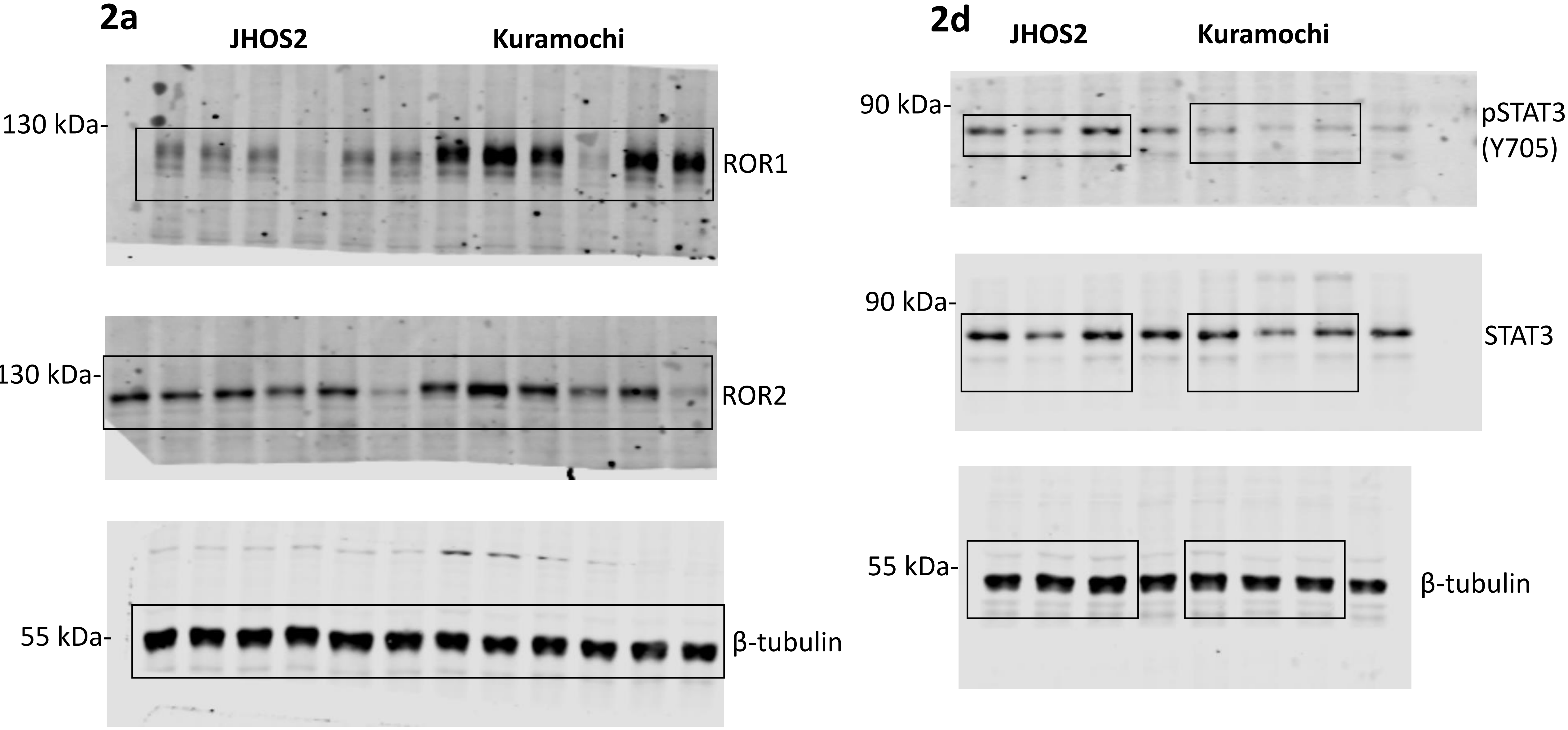

Figure 3c, uncropped WB (JHOS2 cell line)

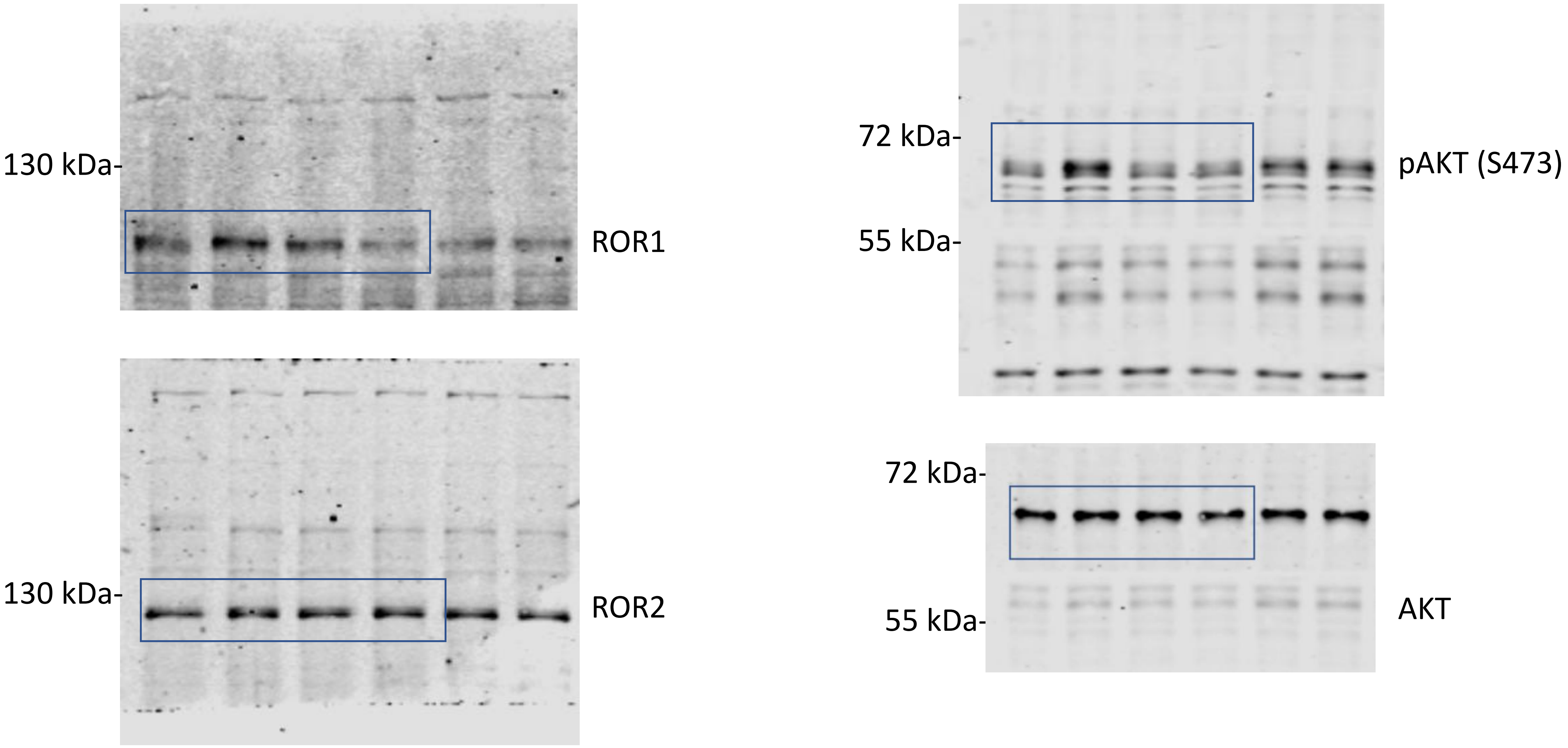

Figure 3c, uncropped WB (JHOS2 cell line)

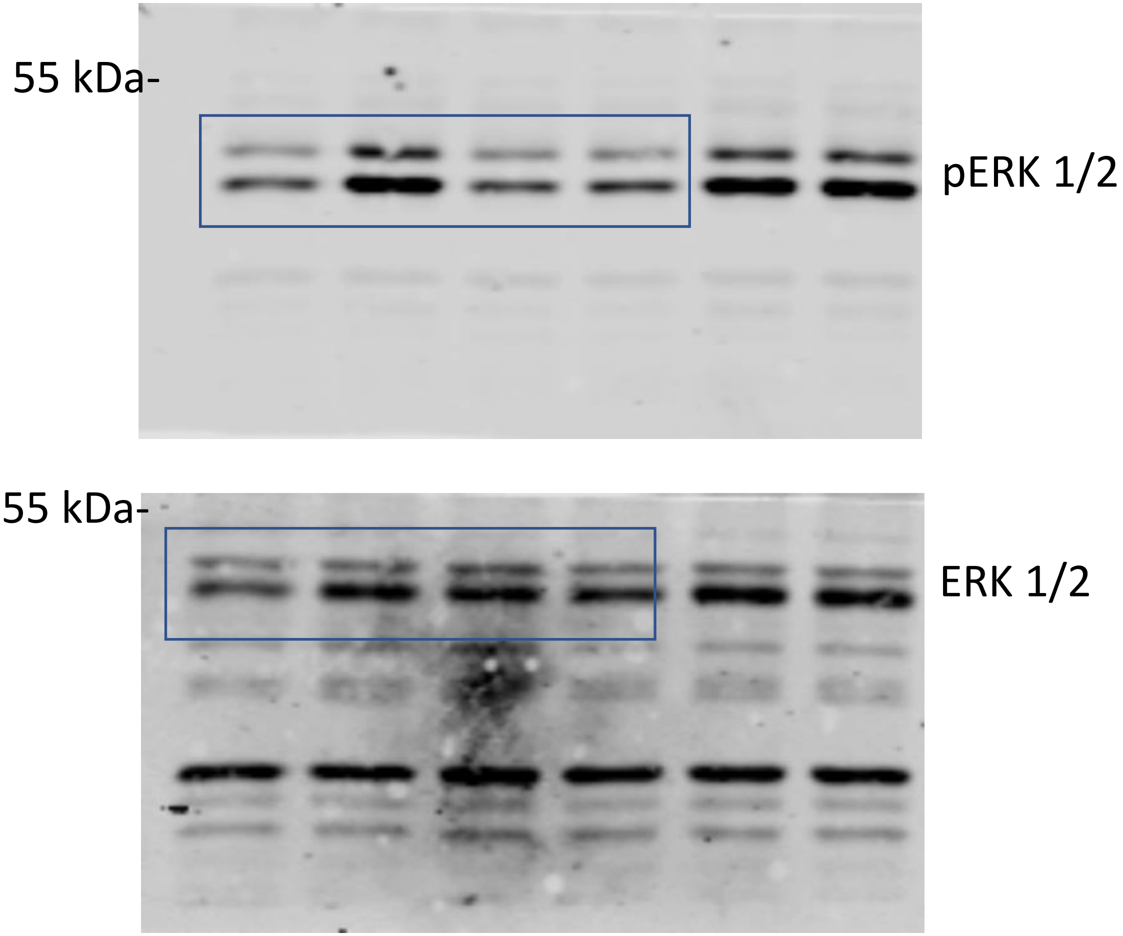

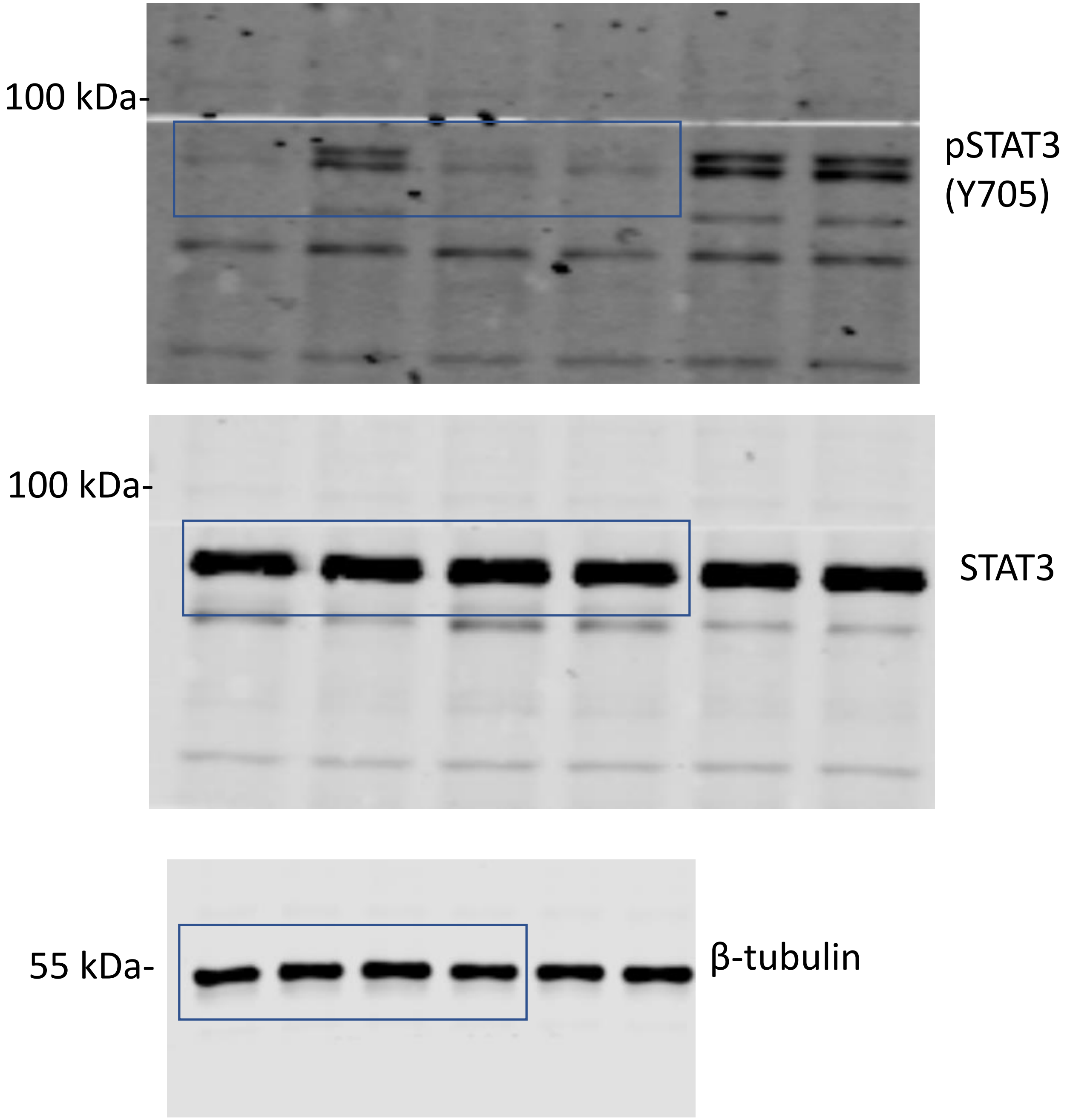

Figure 3c, uncropped WB (Kuramochi cell line)

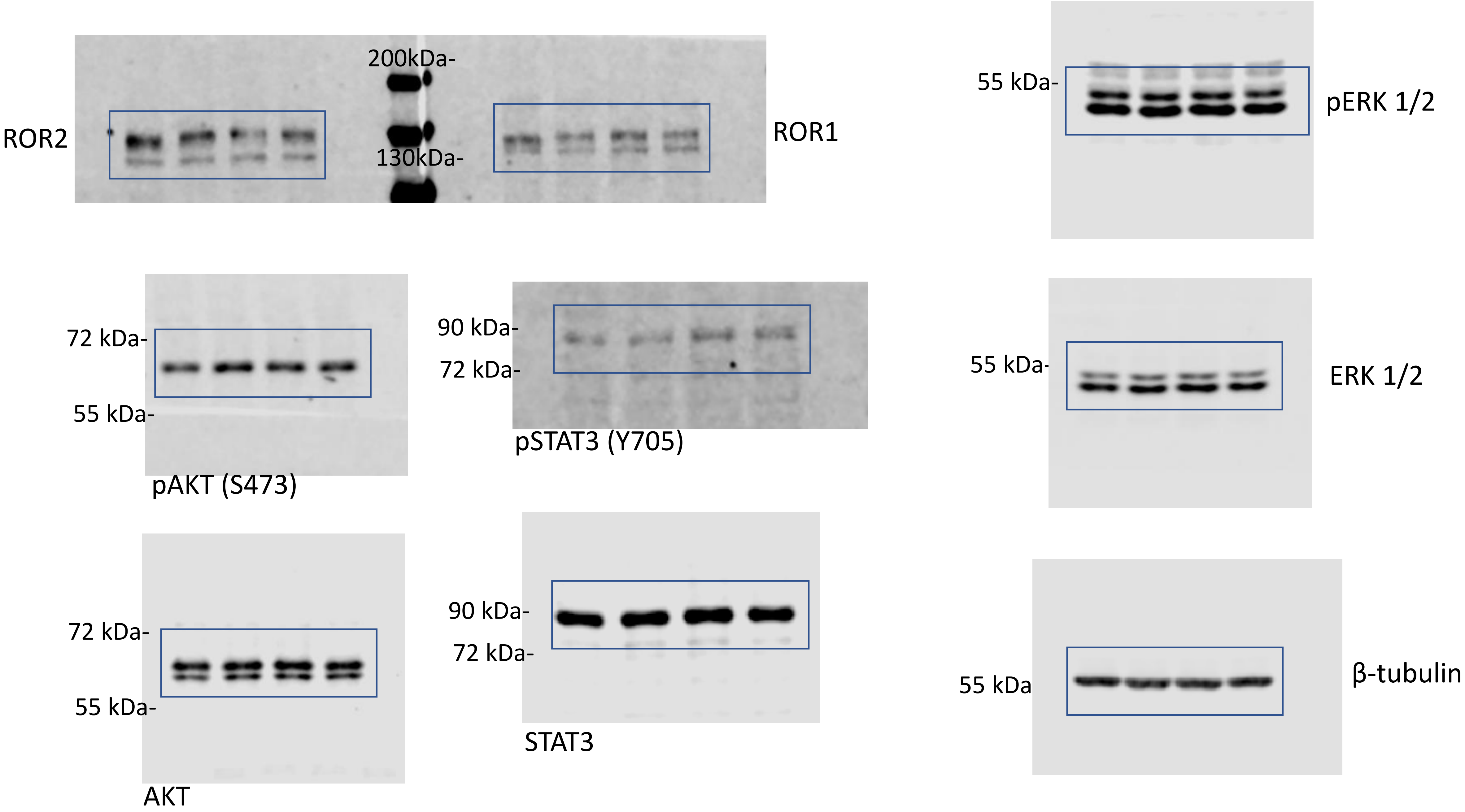

Figure 3d, uncropped WB (JHOS2 cell line)

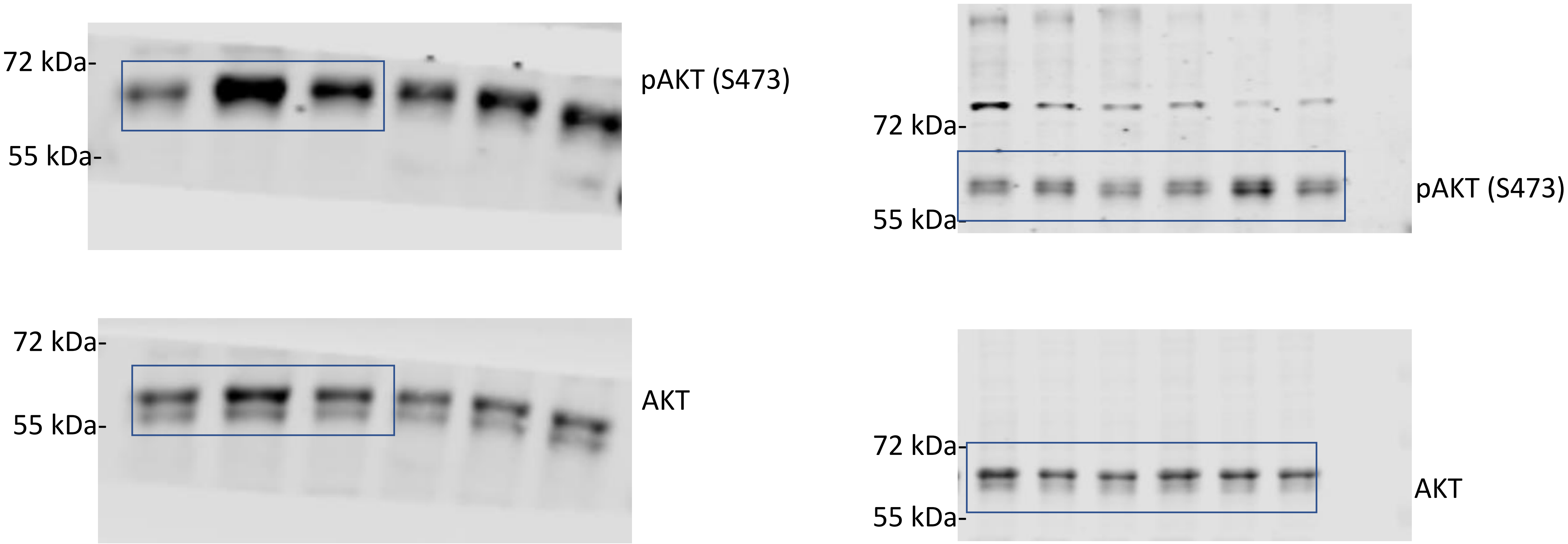

Figure 3d, uncropped WB (JHOS2 cell line)

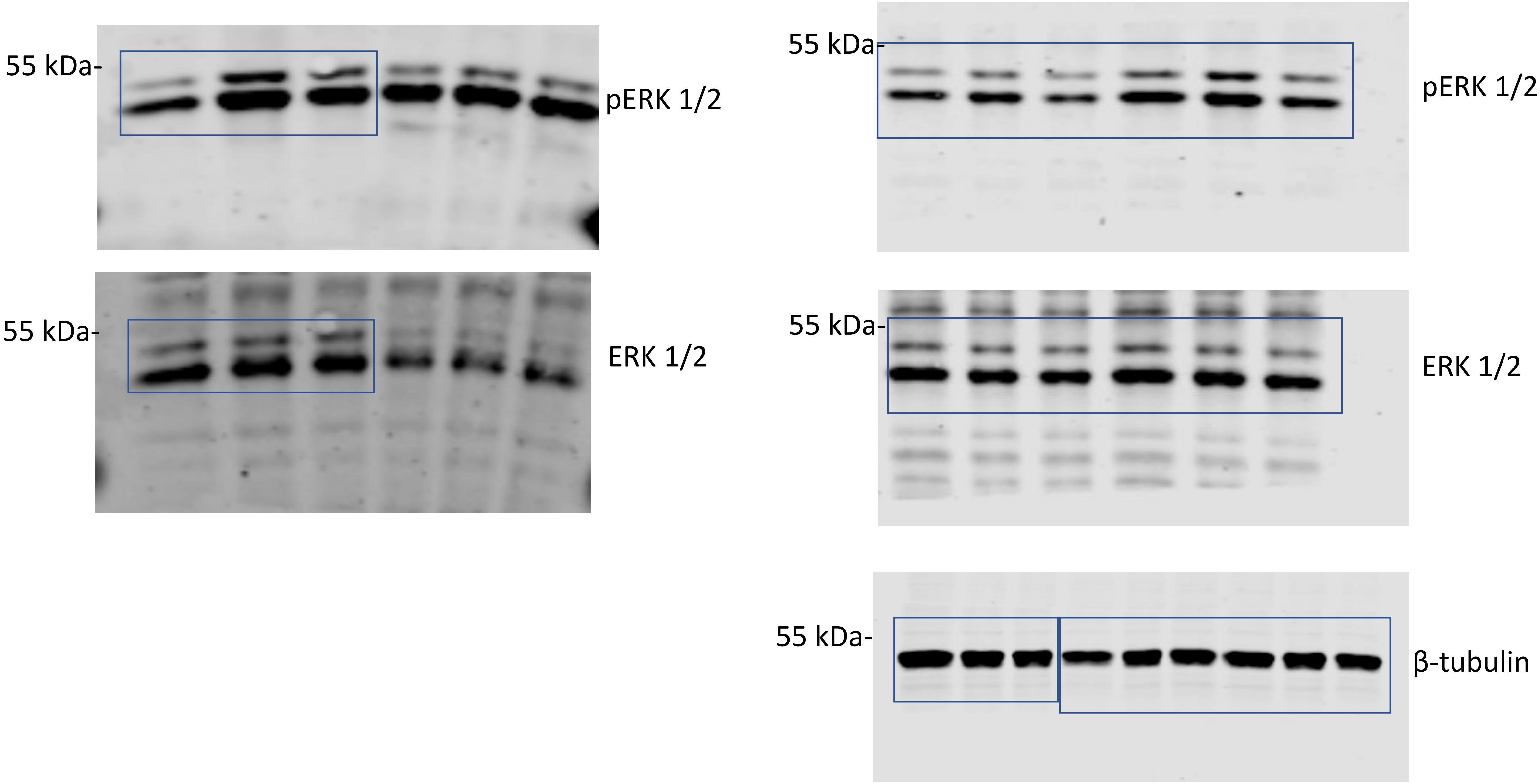

Figure 3d, uncropped WB (Kuramochi cell line)

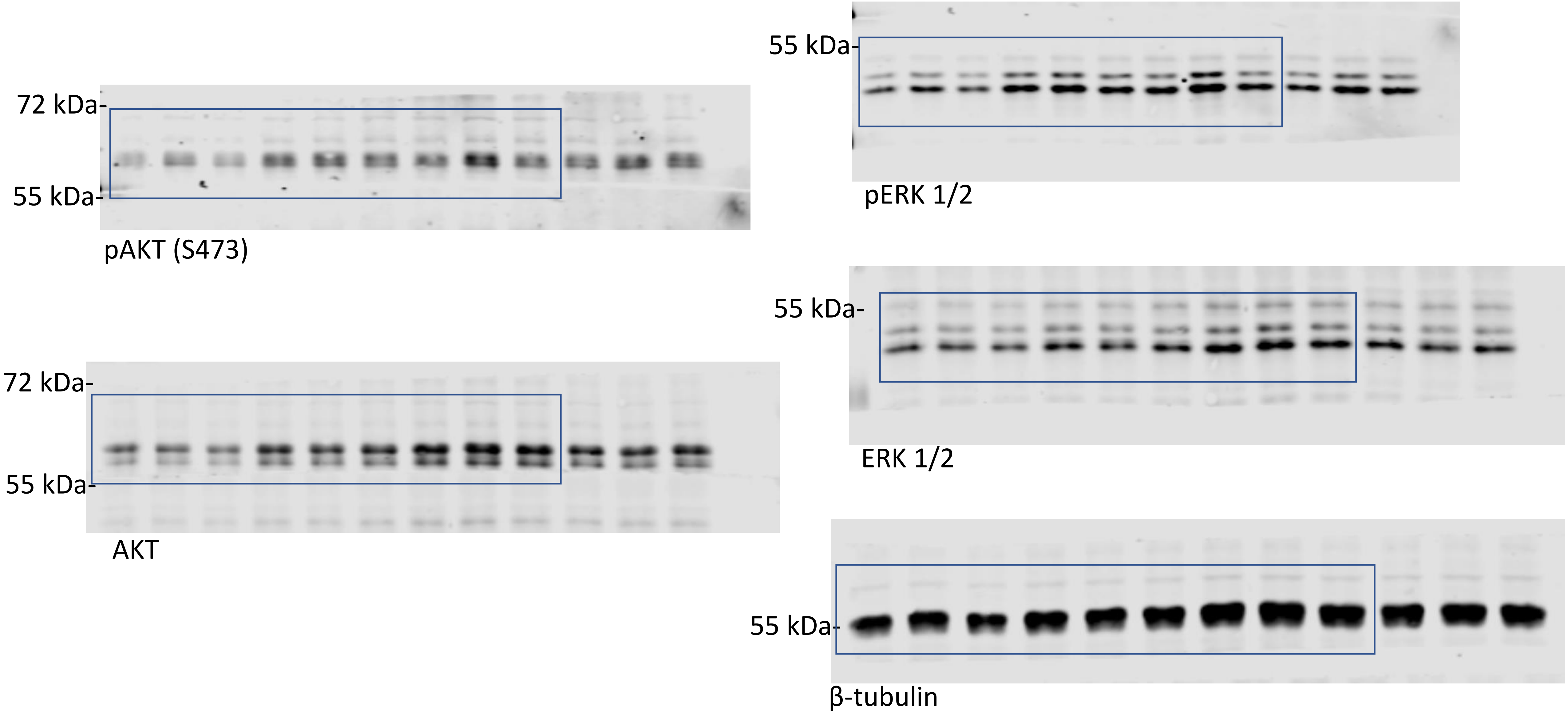

Supplement: Supplementary file 2 — Original Data Files [file 41420_2023_1527_MOESM2_ESM.pdf]
