# Supplementary figures and images for: ROR1-STAT3 signaling contributes to ovarian cancer intra-tumor heterogeneity
Source: Cell Death Discov. 2023 Jul 3;9:222. doi: 10.1038/s41420-023-01527-6 (PMC10317980; doi:10.1038/s41420-023-01527-6)

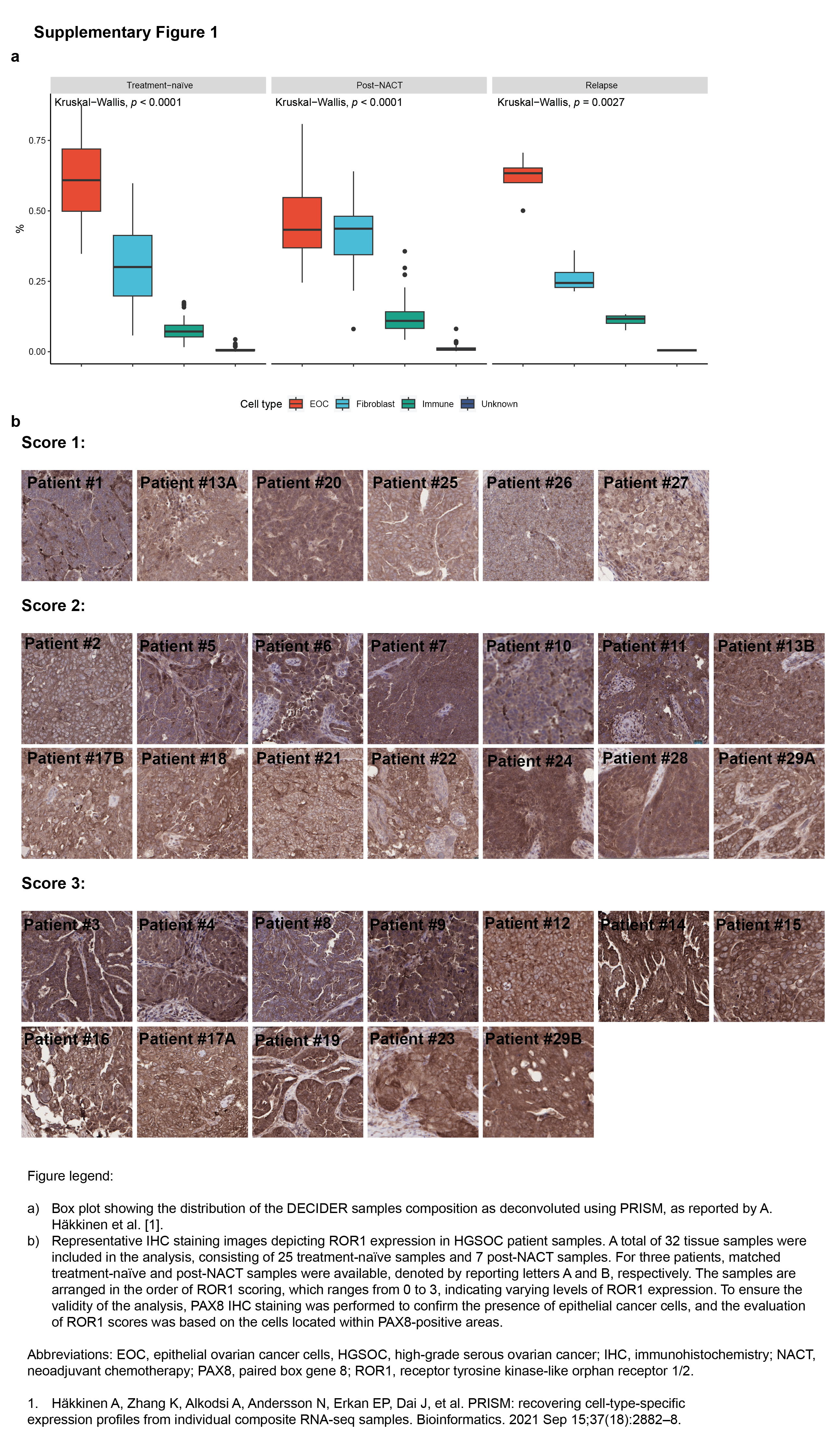

Supplement: Supplementary file 3 — Supplementary Figure 1 [file 41420_2023_1527_MOESM3_ESM.png]

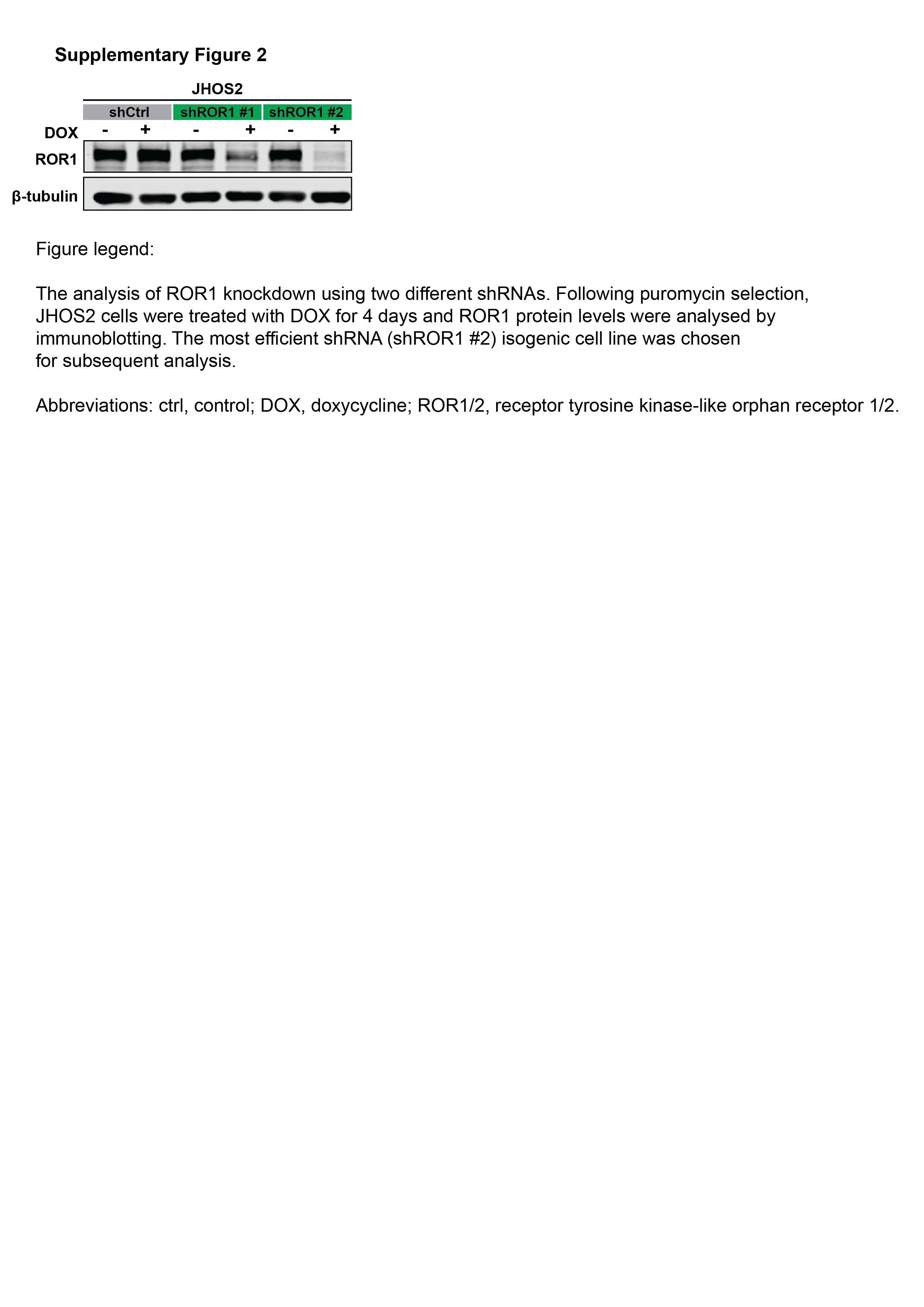

Supplement: Supplementary file 4 — Supplementary Figure 2 [file 41420_2023_1527_MOESM4_ESM.png]

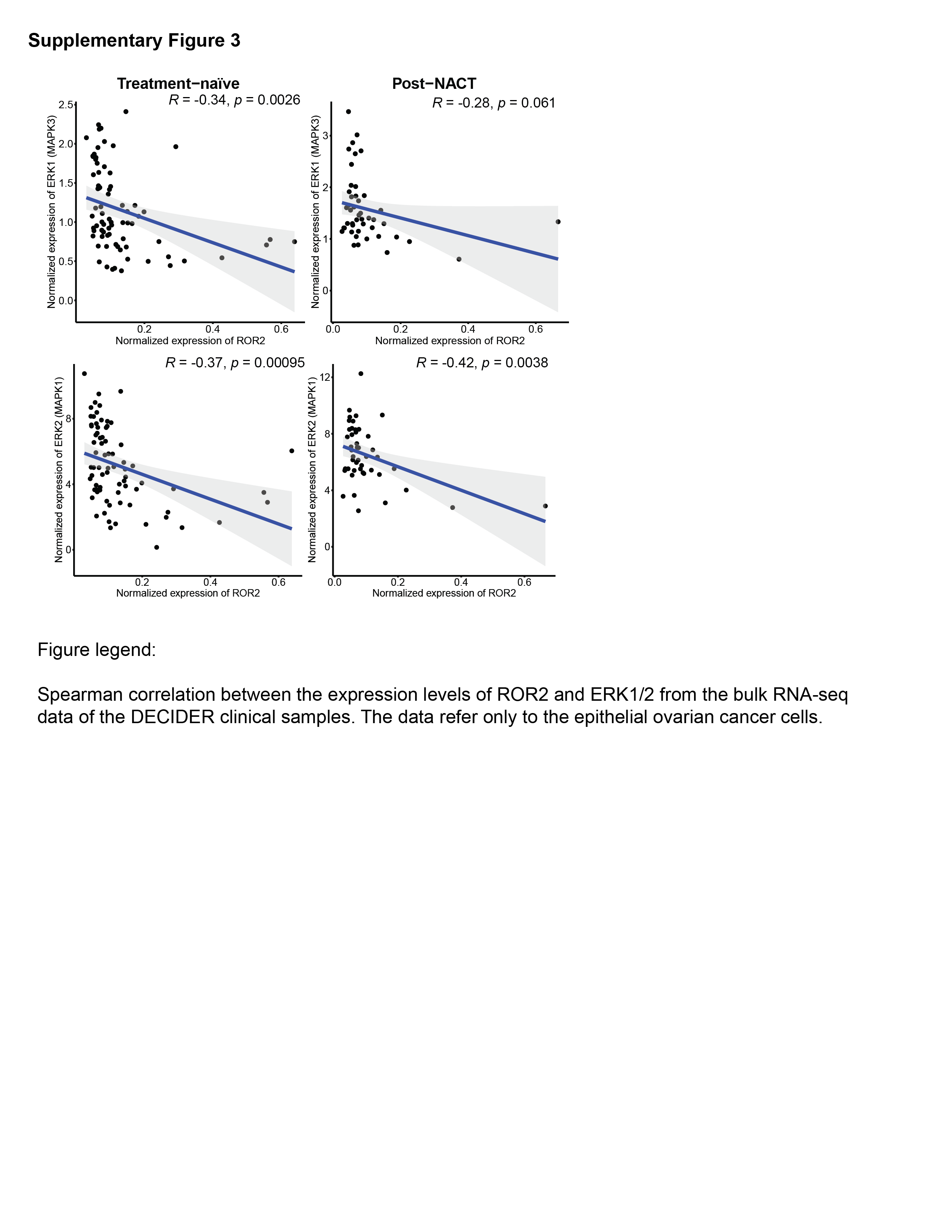

Supplement: Supplementary file 5 — Supplementary Figure 3 [file 41420_2023_1527_MOESM5_ESM.png]

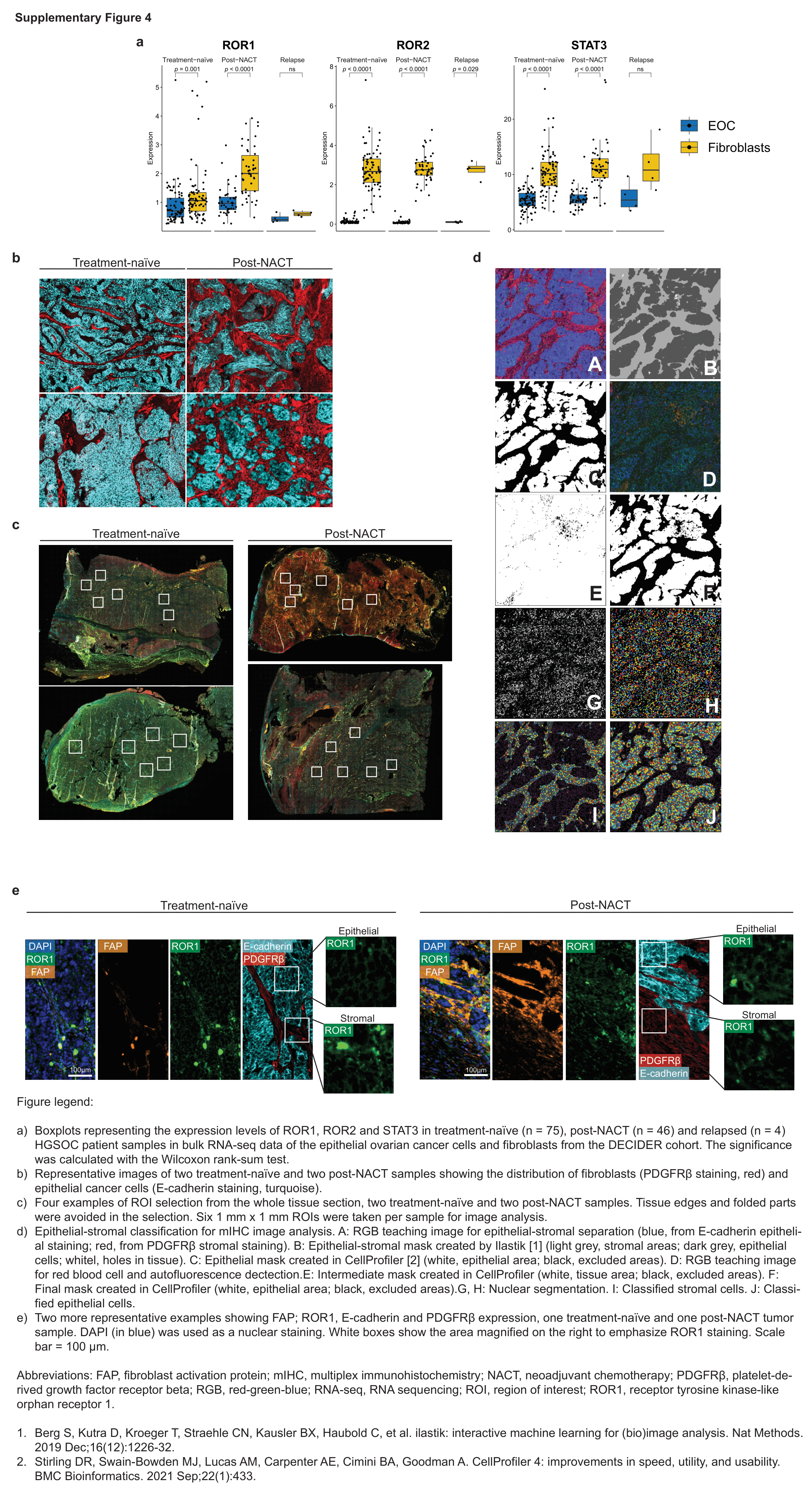

Supplement: Supplementary file 6 — Supplementary Figure 4 [file 41420_2023_1527_MOESM6_ESM.png]

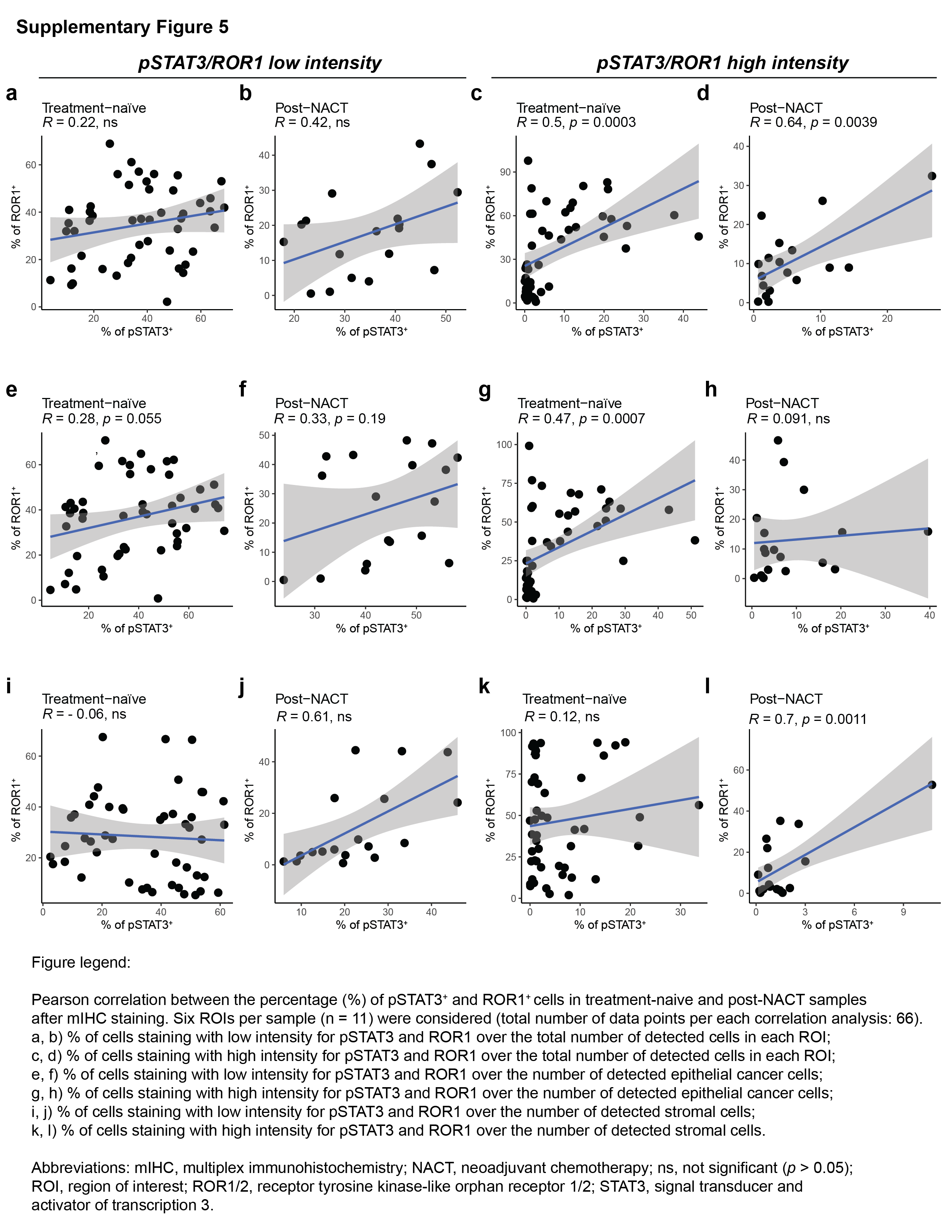

Supplement: Supplementary file 7 — Supplementary Figure 5 [file 41420_2023_1527_MOESM7_ESM.png]

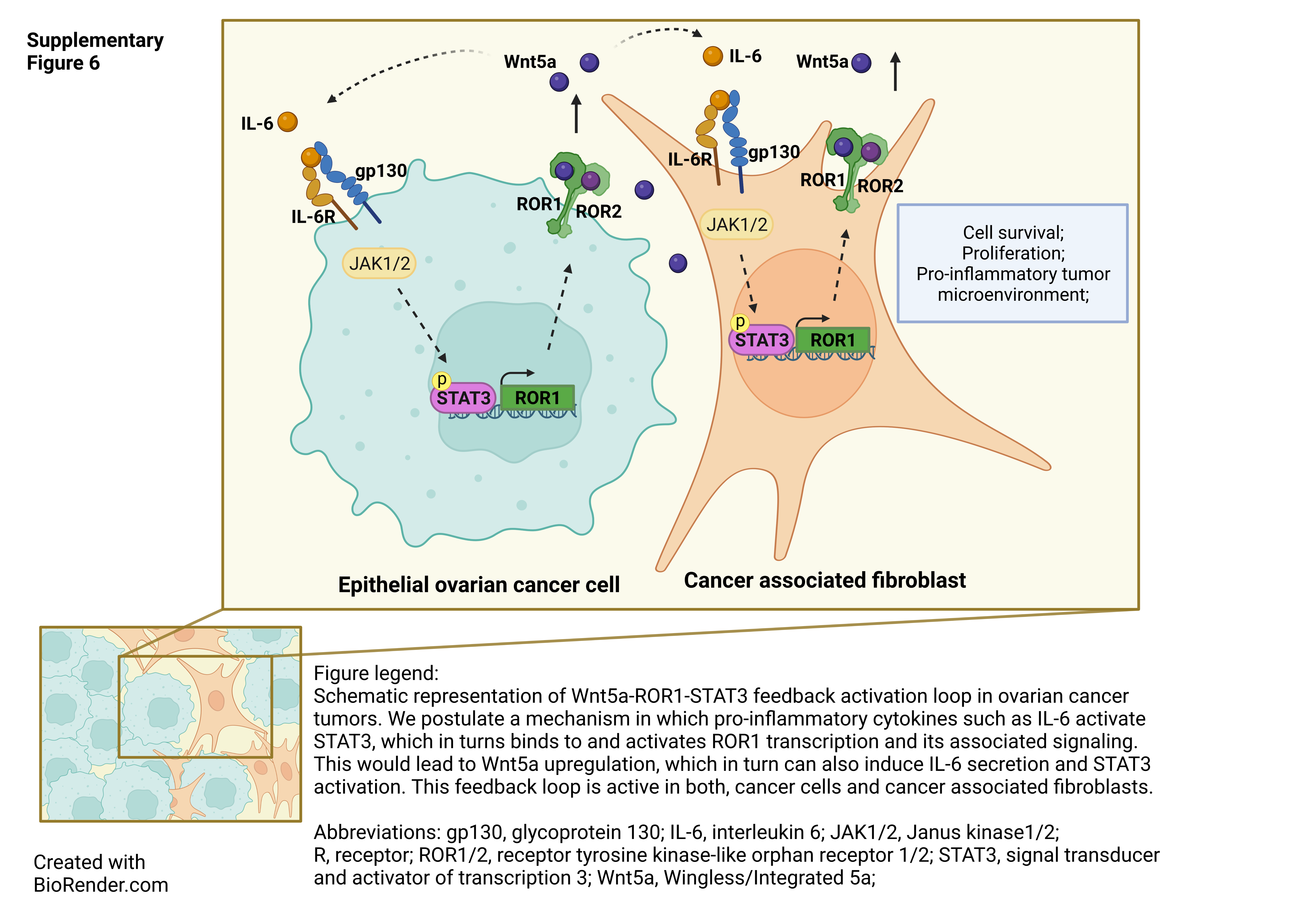

Supplement: Supplementary file 8 — Supplementary Figure 6 [file 41420_2023_1527_MOESM8_ESM.png]
